# Postnatal ontogeny and the evolution of macrostomy in snakes

**DOI:** 10.1098/rsos.160612

**Published:** 2016-11-09

**Authors:** Agustín Scanferla

**Affiliations:** CONICET, Instituto de Bio y Geociencias del NOA (IBIGEO), 9 de Julio N° 14 (A4405BBB), Rosario de Lerma, Salta, Argentina

**Keywords:** snakes, postnatal ontogeny, macrostomy, evolution

## Abstract

Macrostomy is the anatomical feature present in macrostomatan snakes that permits the ingestion of entire prey with high cross-sectional area. It depends on several anatomical traits in the skeleton and soft tissues, of which the elongation of gnathic complex and backward rotation of the quadrate represent crucial skeletal requirements. Here, the relevance of postnatal development of these skull structures and their relationship with macrohabitat and diet are explored. Contrary to the condition present in lizards and basal snakes that occupy underground macrohabitats, elements of the gnathic complex of most macrostomatan snakes that exploit surface macrohabitats display conspicuous elongation during postnatal growth, relative to the rest of the skull, as well as further backward rotation of the quadrate bone. Remarkably, several clades of small cryptozoic macrostomatans reverse these postnatal transformations and return to a diet based on prey with low cross-sectional area such as annelids, insects or elongated vertebrates, thus resembling the condition present in underground basal snakes. Dietary ontogenetic shift observed in most macrostomatan snakes is directly linked with this ontogenetic trajectory, indicating that this shift is acquired progressively as the gnathic complex elongates and the quadrate rotates backward during postnatal ontogeny. The numerous independent events of reversion in the gnathic complex and prey type choice observed in underground macrostomatans and the presence of skeletal requirements for macrostomy in extinct non-macrostomatan species reinforce the possibility that basal snakes represent underground survivors of clades that had the skeletal requirements for macrostomy. Taken together, the data presented here suggest that macrostomy has been shaped during multiple episodes of occupation of underground and surface macrohabitats throughout the evolution of snakes.

## 1. Introduction

Snakes are gape-limited predators that swallow their prey whole, that is, without mechanical reduction through an intraoral treatment prior to ingestion. Among the wide diversity of extant snakes, basal forms such as scolecophidians (worm-like snakes) and basal alethinophidians (pipe snakes, shieldtail snakes) occupy underground macrohabitats and feed on prey of small size and/or small cross-sectional area, such as insects, earthworms and elongated vertebrates. By contrast, alethinophidian snakes included in the clade Macrostomata (pythons, boas, dwarf boas and colubroids) have developed in extreme this feeding strategy ingesting large prey with large cross-sectional area in relation to their head dimensions*.* This particular feeding behaviour present in macrostomatans is possible due to an anatomical feature labelled as macrostomy [1,2], which requires complementary notable modifications in skeletal and soft tissue organs such as increased length of the gnathic complex (palatomaxillary arch, suspensorium and mandible) and modifications of the intermandibular soft tissues to allow stretching [1–7]*.* Macrostomy permits exploiting an enormous diversity of prey types, a fact that has deep implications for the occupation of diverse macrohabitats and evolutionary success of macrostomatan snakes [1]. The acquisition of this astonishing anatomical feature results in one of the most interesting innovations in vertebrate feeding, but much of the evolutionary events that shaped macrostomy remain uncertain.

Studies concerning postnatal ontogeny have become a powerful source of information about evolutionary processes in the skeleton of squamates [8,9]. However, transformations during postnatal ontogeny in snakes remain largely unknown. To date, the few studies of snake postnatal development [10–13] have identified that skull components directly involved in macrostomy experience important changes during postnatal ontogeny. Here, I analyse postnatal transformations in the skull of several representatives of major groups of snakes to investigate the impact of postnatal ontogeny on the evolution of cranial requirements for macrostomy. In order to explore the relation between macrohabitat, diet and skull morphology, I also perform an exhaustive survey comparing these variables in all major groups of living snakes to evaluate their relationship in the evolution of macrostomatan snakes. Finally, I place these results in a phylogenetic context to trace the evolution of macrostomy in the context of the evolution of snake feeding.

## 2. Material and methods

### 2.1. Specimens examined

Postnatal transformations were examined in dry and cleared and stained skulls of several lizard and snake groups (electronic supplementary material). Most specimens were studied first hand, but in other cases, I relied on photographs or published descriptions of embryonic and postnatal series. Postnatal sequences utilized represent series of growth stages assembled with available specimens in collections.

### 2.2. Quantitative analysis

Several cranial bones were measured in selected representatives of the major clades of snakes and simply quantified as ratios and figured with respect to the snout--condyle length, a standard measure that corresponds with the longitudinal measurement of the skull (see electronic supplementary material). Small specimens were measured to the nearest 0.1 mm using an ocular micrometer.

### 2.3. Feeding morphological–ecological survey

To explore the relationship between the morphological and ecological traits of snake feeding, I surveyed information about skull anatomy, body size (snout--vent length, SVL), macrohabitat and prey type in 147 species representing all major groups of extant snakes (electronic supplementary material, S2). In order to standardize terminology, I first defined accurately each term used in reference to underground (fossorial, cryptozoic) and surface (terrestrial, arboreal and aquatic) macrohabitats and diet types (electronic supplementary material, S1 and S2). This survey is exhaustive with respect to the currently recognized major clades of snakes, including nearly all basal and advanced alethinophidians that occupy underground macrohabitats. Information concerning body size, macrohabitat and prey items was obtained using published studies about natural history of selected species (see electronic supplementary material). In the particular case of food habits, I chose only studies based on gut content of a large number of individuals, with the exception of a few relevant taxa without specific studies about their diet (e.g. *Xenopeltis*), which were scored using studies that register single records of predation or gut contents. In order to test for significant differences in these traits throughout the phylogeny, I performed a phylogenetic MANOVA [14] using the R package ‘Geiger’ [15]. A phylogeny of extant snakes was taken from the maximum-likelihood phylogenetic tree of Pyron *et al.* [16].

## 3. Results

### 3.1. Postnatal transformations in gnathic complex

#### 3.1.1. Elongation of the palatomaxillary arch, suspensorium and mandible

**Figure 1.**
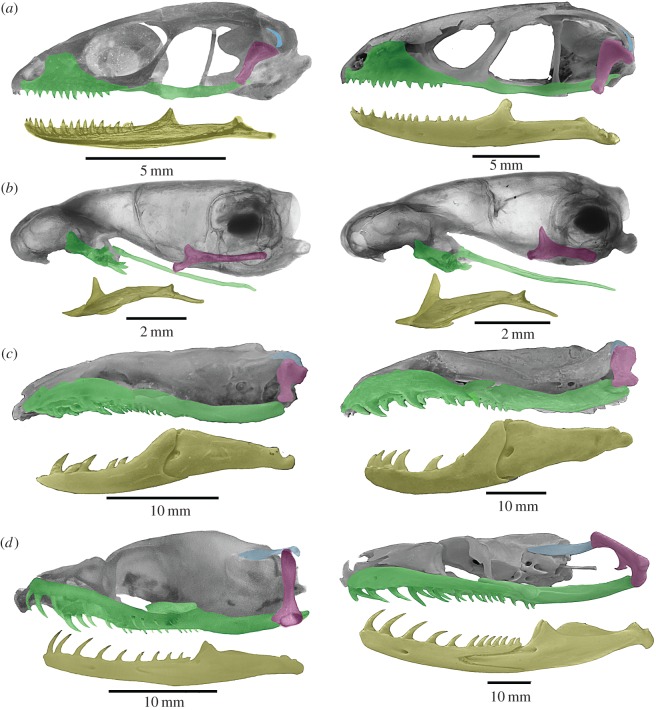
Postnatal transformations in the gnathic complex in lizards and snakes. (*a*) Juvenile (SVL 56 mm) and adult (SVL 237 mm) of the anguid lizard *Ophiodes intermedius*; (*b*) juvenile (SVL 43 mm) and adult (SVL 131 mm) of the typhlopid scolecophidian snake *Amerotyphlops brongersmianus*; (*c*) juvenile (SVL 212 mm) and adult (SVL 895 mm) of the basal alethinophidian snake *Anilius scytale*; (*d*) juvenile (SVL 311 mm) and adult (SVL 1620 mm) of the macrostomatan snake *Boa constrictor*.

With increasing postnatal stage, the elements that form the gnathic complex of basal snakes such as scolecophidians, basal alethinophidians and basal macrostomatans (*Xenopeltis*, *Loxocemus*) experience isometric growth with respect to the rest of the skull (figure 1*b,c*; electronic supplementary material, S1 and figure S2), similar to the condition previously described for lizards [17–21]. As in lizards, relative dimensions of the pterygoid, supratemporal, quadrate and mandible of basal snakes increase their length isometrically with respect to the skull length during postnatal growth (figures 1*a–c* and 3). However, the posterior elongation of the pterygoid, supratemporal and mandible never surpasses the posterior boundary delimited by the occipital–vertebral joint. Also, the quadrate bone grows with the same isometric pattern of the rest of the gnathic elements.

**Figure 2.**
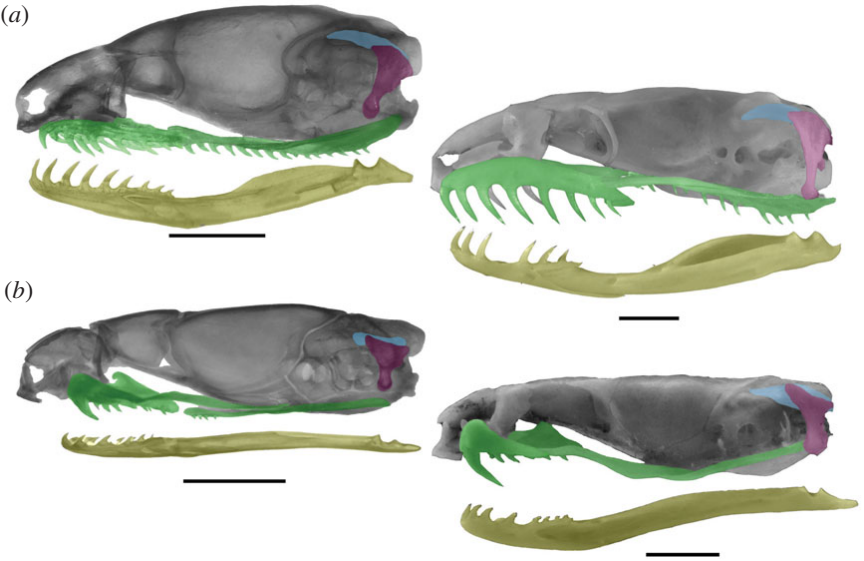
Postnatal transformations in the gnathic complex of cryptozoic macrostomatans. (*a*) Lateral view of the skull of a juvenile (SVL 114 mm) and adult (SVL 277 mm) of the dipsadine colubroid *Atractus reticulatus*; (*b*) juvenile (SVL 182 mm) and adult (SVL 921 mm) of the elapid *Micrurus pyrrhocryptus*. Scale bars, 2 mm.

**Figure 3.**
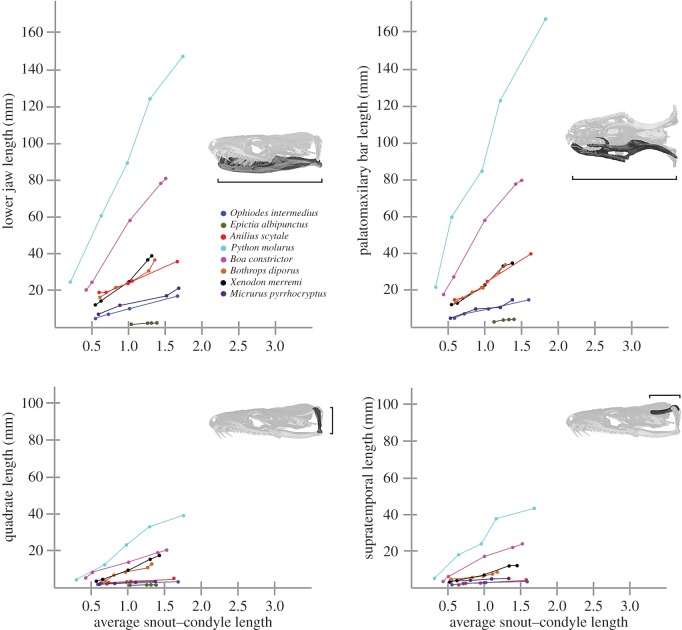
Metrics of changes in underground and surface snake gnathic complex elements during postnatal ontogeny.

By contrast, the major changes that occur in the skull of most macrostomatan snakes from hatchling to adult stages comprise the allometric elongation of the elements that form the gnathic complex with respect to the rest of the skull (figures 1*d* and 3; electronic supplementary material, S1 and figure S1). This growth is clearly evidenced by the elongation of the quadrate ramus of the pterygoid bone, supratemporal and mandible beyond the posterior limit marked by the occipital–vertebral joint. The posterior elongation of the supratemporal bone generates the free-ending process, which represents one of the most distinctive traits of the macrostomatan skull. The length of the gnathic complex varies considerably among macrostomatan subclades, and it tends to be more slender in snakes with moderate to large SVL (more than 1500 mm). The quadrate bone also shows a significant growth through the elongation of the quadrate shaft. Notably, some groups of macrostomatans, specifically small ones, are an exception because these small snakes exhibit isometric growth of the gnathic complex in relation to skull length, resembling the condition present in lizards and basal snakes (figures 2 and 3; electronic supplementary material, S1 and figure S3). This shift involves the reversion of key characters for macrostomy like the shortening or absence of the free-ending process of the supratemporal bone and shortening of quadrate bone, resulting in a stout quadrate that displays a strikingly similar shape to that observed in basal alethinophidians.

#### 3.1.2. Quadrate rotation

The quadrate of macrostomatan snakes experiences a noteworthy backward rotation process during embryonic development [22–25]. In late embryonic stages, the ossifying quadrate undergoes a counter-clockwise rotation to a vertical position, which is typical of postembryonic stages in basal alethinophidians, basal macrostomatans and hatchling individuals of core macrostomatans. This rotatory process seems to be absent in scolecophidians because the quadrate bone is rostroventrally slanted in all postnatal stages [26].

**Figure 4.**
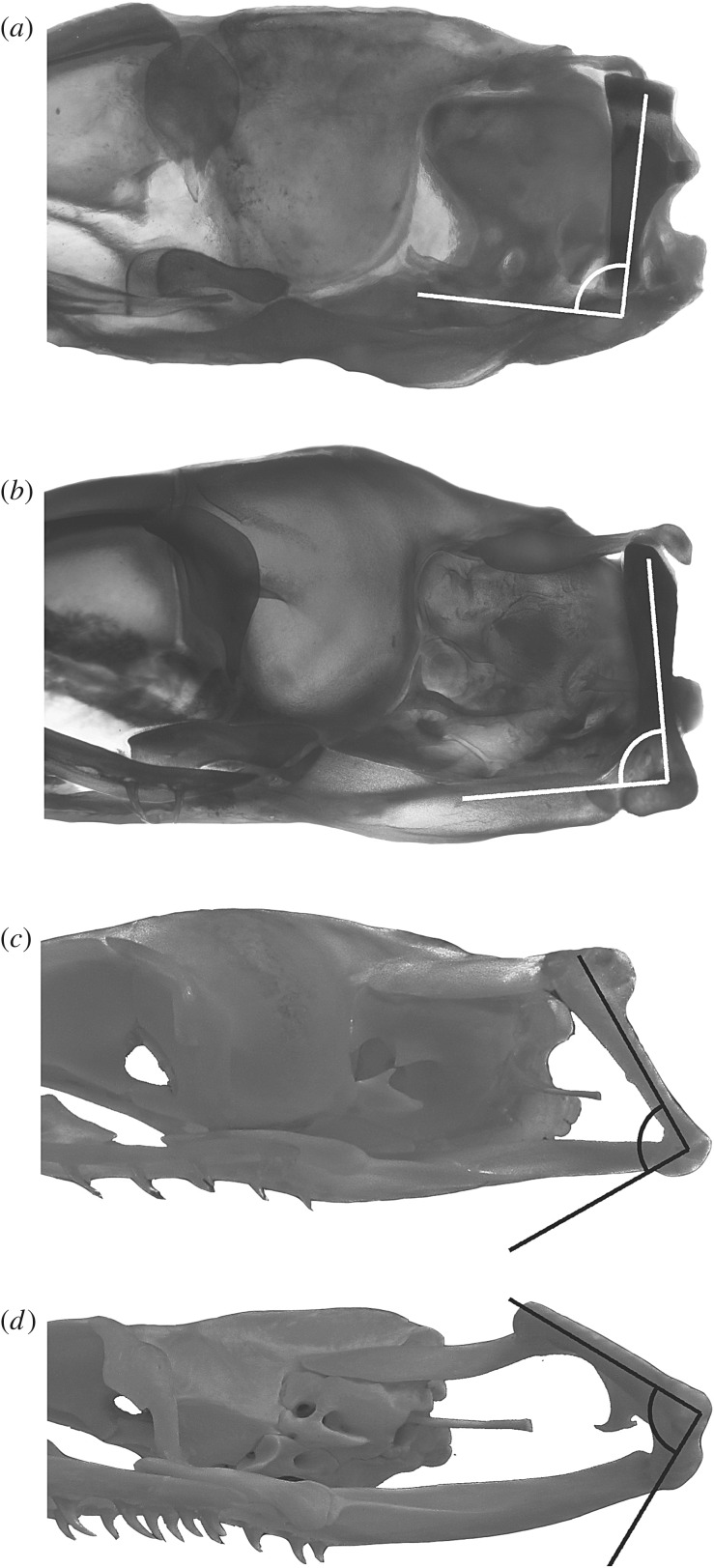
Rotation of the quadrate bone during postnatal development in the macrostomatan snake *Boa constrictor*. (*a*) Late embryo (SVL 299 mm); (*b*) hatchling (SVL 311 mm); (*c*) juvenile (SVL 463 mm); (*d*) adult (SVL 1620 mm). Not to scale.

Remarkably, adults of most macrostomatans exhibit a further backward inclined position of the quadrate with respect of the vertical or slightly inclined position exhibited by hatchings (figure 1*d*; electronic supplementary material, S1 and figure S4). Available ontogenetic series indicate that this position is the result of the continuation during the postnatal development of the rotatory process initiated in embryonic stages. Late embryos and postnatal individuals of the macrostomatan *Boa constrictor* (figure 4) show this condition, where the quadrate exhibits an intermediate rotation process in a vertical position with respect of the rest of the skull. During subsequent postnatal stages this backwards rotation continues, reaching the caudoventral inclined orientation that characterizes adult individuals (figure 4*d*; electronic supplementary material, S1 and figure S4). It is important to note that macrostomatans without the elongation of the gnathic complex also lack the postnatal rotation process of the quadrate bone (figure 2; electronic supplementary material, S1 and figure S3).

### 3.2. A comparative survey among morphological and ecological traits

The performed comparative survey supports previous observations [1,27] that claimed a close relationship between morphological and ecological traits concerning snake feeding.

**Figure 5.**
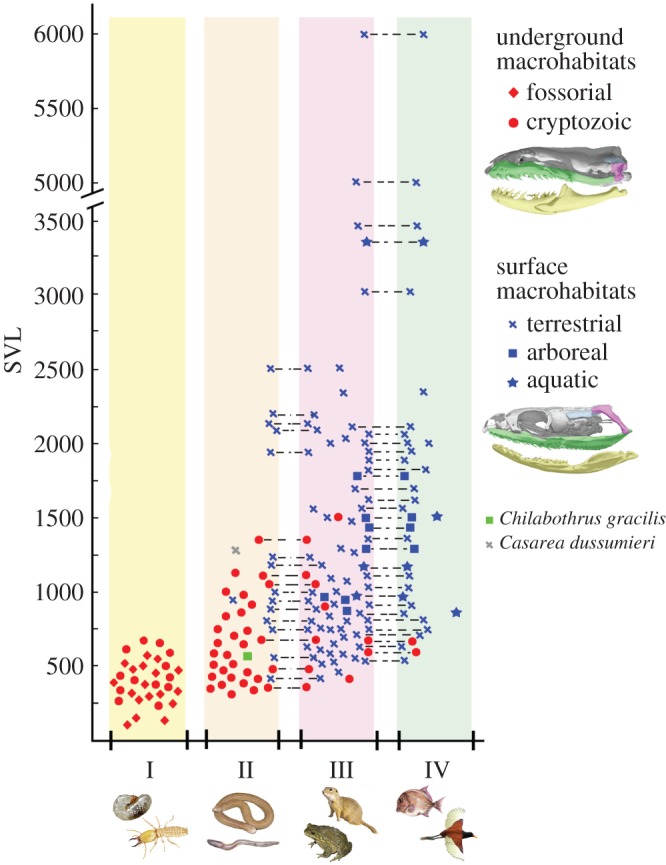
Graphical summary of the ecological morphological survey performed between body size (SVL), prey type and macrohabitat preferences of a set of adult extant snakes. Each point represents one species and points joined by a dotted line represent a single species with two prey type preferences.

Basal groups (scolecophidians, basal alethinophidians), which exploit underground macrohabitats (fossorial, cryptozoic), exhibit a small gape size due to their short gnathic complex and a rostroventrally or vertically oriented quadrate (figure 5; electronic supplementary material, S2). Almost invariably, these small-sized (SVL < 1000 mm) underground species ingest small prey with a small cross-sectional area such as insects, earthworms or elongated vertebrates. Remarkably, macrostomatan species that occupy underground macrohabitats, which also lack the postnatal changes in gnathic complex, mirror basal snakes in these feeding aspects. Results of the phylogenetic MANOVA analysis indicate there are significant differences in prey type and macrohabitat depending on SVL (*F* = 8.274 3, 126 *p* = 0.00429) and do not simply reflect the phylogenetic structure of the species sample. In other words, small underground snakes, independent of their phylogenetic position, lack postnatal transformations in gnathic complex and consume prey with small cross-sectional area throughout their whole lifespan.

Nearly all snakes that occupy surface macrohabitats (terrestrial, aquatic and arboreal) belong to the clade Macrostomata (figure 5; electronic supplementary material, S2). These snakes display the postnatal elongation of the gnathic complex and the quadrate rotation, and thus acquire in adult stages a large gape size that allows the ingestion of an extraordinary array of large prey with a large cross-sectional area. However, juvenile surface macrostomatans exhibit a small gape size owing to their early postnatal stage, consuming prey with small cross-sectional area. Notably, adult individuals of the few small macrostomatan species that occupy surface macrohabitats and were postulated as paedomorphic forms (e.g. *Casarea dussumieri, Chilabothrus gracilis*) lack the postnatal changes of the gnathic complex, and also consume prey items with a small cross-sectional area (figure 5; electronic supplementary material, S2).

## 4. Discussion

### 4.1. Postnatal ontogeny and dietary shift

It is widely acknowledged that most macrostomatan snakes experience an ontogenetic dietary shift, which implies a dietary change from small ectothermic prey with a small cross-sectional area as juveniles to bulky often endothermic prey with large cross-sectional area as adults. This shift necessarily implies an increase in gape size and other changes in behavioural and physiological characteristics [1,28–30]. Here, I argue that crucial factors for gape size such as length of components of gnathic complex and quadrate orientation undergo dramatic changes during postnatal ontogeny in macrostomatans. Hence, postnatal changes experienced by the gnathic complex constitute intrinsic anatomical factors that lead the ontogenetic dietary shift. Accordingly, the ability to ingest prey with large cross-sectional area is acquired progressively as lengthening of gnathic complex and backward quadrate rotation occurs during the course of postnatal ontogeny of surface macrostomatan snakes. Underground snakes, as well as the few surface macrostomatans that lack these changes during skull postnatal ontogeny, do not experience an ontogenetic dietary shift, thus highlighting the relevance of postnatal changes in the gnathic complex described here to the achievement of an ontogenetic dietary shift.

### 4.2. Macrostomy and ‘regressed macrostomatans'

Miniaturization, which is a process usually attributed to paedomorphosis, is frequently correlated with burrowing habits in squamate reptiles [31–33]. This process generates shortening of the gnathic complex and verticalization of the quadrate bone in cryptozoic macrostomatans [34], and thus a regression of the crucial osteological requirements for macrostomy analysed herein. Based on the results of this study, the effects of miniaturization in the gnathic complex of cryptozoic macrostomatans can be inferred to have arisen through the truncation of the postnatal ontogenetic trajectory described above. Taking into account that at least 10 distantly related subclades of cryptozoic macrostomatan snakes lost skeletal requirements for macrostomy independently (electronic supplementary material, S1 and figure S5), this phenomenon appears to be recurrent in extant clades of snakes that occupy underground macrohabitats. Furthermore, recent molecular and combined phylogenetic analyses [16,35–37] yielded intriguing results where macrostomatan snakes with well-developed cranial requirements for macrostomy are nested within basal alethinophidians that lack these requirements and vice versa. Taken together, this body of information indicates that loss and reappearance of cranial requirements for macrostomy could have occurred several times in the history of snakes. Thus, scolecophidians and basal alethinophidians could be considered as miniaturized underground survivors (‘regressed macrostomatans’) of clades that formerly occupy surface macrohabitats as was discussed recently [26,38,39]. Although this hypothesis clashes with the traditional perspective, according to which the structure of the gnathic complex in basal snakes is ancestral, the presence of skeletal requirements for macrostomy in non-macrostomatan fossil snakes with large body size supports this interpretation. The stem snake *Dinilysia patagonica* exhibits a postnatal growth pattern of the supratemporal and lower jaws that positioned the mandibular articulation beyond the posterior limit marked by the occipital–vertebral joint [13]. Also, cranial requirements for macrostomy are present in Cretaceous marine simoliophiid snakes [40–42], a clade that is positioned outside Macrostomata in many phylogenetic analyses [36,37,43–45]. Moreover, pycnodontiform fish remains were reported as a gut content in the simoliophiid *Pachyrhachis problematicus* [40,46], a kind of prey that certainly represented a challenge for feeding because these fishes had deep, rounded and laterally compressed bodies.

### 4.3. The evolution of macrostomy

**Figure 6.**
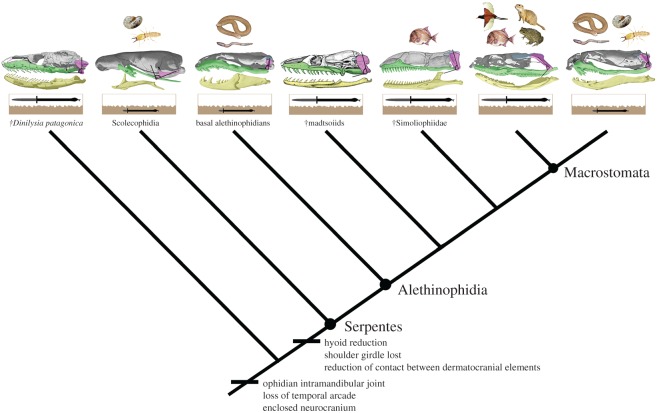
Simplified phylogenetic tree of snakes [45] showing the information discussed in this work about body size, macrohabitat, prey type preferences and osteological requirements for macrostomy. Note the early appearance of crucial osteological requirements for macrostomy and the elongation of the gnathic complex present in non-macrostomatan simoliophiids.

The overwhelming majority of living snake species belong to Macrostomata, making them one of the most diverse lineages of extant reptiles. Thus, the ability to consume entire bulky prey allowed by macrostomy arguable constitutes the most relevant key innovation that has fuelled their evolutionary success. Beyond the anatomical particularities of the gnathic complex analysed above, there are other skeletal transformations necessary to achieve this astonishing feeding behaviour, such as an intramandibular joint, reduction of bony contact between skull elements to permit unilateral movements of the upper jaw, loss of contact between hyoid and skull, an enclosed neurocranium, loss of the temporal arcade and loss of contact between ribs with sternum [1–4]. Remarkably, pressures imposed by underground macrohabitats have been postulated as triggers of most of these skeletal transformations via heterochronic processes and body elongation [31,34,47,48]. Therefore, underground macrohabitats in the early history of snakes were necessary to acquire a highly kinetic skull and reduce constraints for the transport of entire bulky prey into the stomach. Additionally, a large digestive tract to accommodate entire large prey was probably acquired during this early underground phase [49]. Given the findings of this research, it appears that a surface phase was crucial for releasing the constraints imposed by underground macrohabitats and permitting the elongation of the gnathic complex and the backward rotation of the quadrate bone during postnatal growth to expand gape size for exploitation of large prey. Thus, different episodes of occupation of underground and surface macrohabitats coupled with changes in behaviour and diet have shaped the skull morphology of macrostomatan snakes (figure 6). Regrettably, the appearance of other relevant transformations related to macrostomy such as stretching capabilities and reorganization of intermandibular tissues and adductor musculature are harder to explain with the available data. However, the retention of a well-developed aponeurotic system in the jaw adductor musculature of cryptozoic colubroids and its reduction observed during the postnatal development in terrestrial forms [50] invites consideration of the idea that the evolution of anatomical systems other than the skeleton have been also driven by the alternation of underground--surface macrohabitat occupation.

Finally, the growing fossil record of snakes reveals that non-macrostomatan snakes with the capacity to consume large prey have existed [51,52], and that other species of miniaturized macrostomatan snakes have lost the skeletal requirements for macrostomy [45], thus indicating that the diversity of snake feeding anatomy was more complex than previously thought. Further investigations of the postnatal trajectory of other anatomical systems involved in macrostomy and more efforts towards the discovery of fossil snakes surely will shed light on this captivating evolutionary issue.

## Supplementary Material

Electronic supplementary material S1: Specimens examined, macrohabitat and prey type definitions, morphometric data, phylogenetic MANOVA results, supplementary figures, and references for electronic supplementary materials

## Supplementary Material

Electronic supplementary material S2: summary of diet, prey type, body size and macrohabitat data for snake species included in the comparative survey

## Data accessibility

Electronic supplementary material S1: specimens examined, macrohabitat and prey type definitions, morphometric data, phylogenetic MANOVA results, supplementary figures and references for electronic supplementary materials. Electronic supplementary material S2: summary of diet, prey type, body size and macrohabitat data for snake species included in the comparative survey. All electronic supplementary materials are available from the Dryad Digital Repository http://dx.doi.org/10.5061/dryad.1g626 [53].
