# Supplementary material for: Postnatal ontogeny and the evolution of macrostomy in snakes
Source: R Soc Open Sci. 2016 Nov 9;3(11):160612. doi: 10.1098/rsos.160612 (PMC5180150; doi:10.1098/rsos.160612)
Supplement: Electronic supplementary material S1: Specimens examined, macrohabitat and prey type definitions, morphometric data, phylogenetic MANOVA results, supplementary figures, and references for electronic supplementary materials [file rsos160612supp1.pdf]

Electronic supplementary material for:

Postnatal ontogeny and the evolution of macrostomy in snakes

Agustín Scanferla

CONICET. Instituto de Bio y Geociencias del NOA (IBIGEO), 9 de Julio Nº 14,

Rosario de Lerma, Salta, Argentina. agustin_scanferla@yahoo.com.ar

CONTENTS:

A. Specimens examined 2

B. Quantitative analysis 9

C. Macrohabitat definitions 11

D. Prey types 18

E. Phylogenetic MANOVA analysis 22

F. Supplementary figures 24

G. References for electronic supporting materials (including literature cited in Table S1) 29

A. Specimens examined

Institutional Abbreviations

AMNH, American Museum of Natural History, New York; NHMUK, British Museum of Natural History, London; CAS, California Academy of Science, San Francisco; CENAI, Centro Nacional de Investigaciones Biológicas (currently housed in MACN), Buenos Aires; CM, Carnegie Museum of Natural History, Pittsburgh; FML, Fundación Miguel Lillo, Tucumán; FMNH, Field Museum of Natural History, Chicago; IB, Instituto Butantan, Sao Paulo; LSUMZ, Louisiana State University Museum of Zoology, Baton Rouge; MACN, Museo Argentino de Ciencias Naturales "Bernardino Rivadavia", Buenos Aires; MCN, Museo de Ciencias Naturales de Salta, Salta; MECN, Museo Ecuatoriano de Ciencias Naturales, Quito; MLP, Museo de La Plata, La Plata; MNHN, Museum National d´Histoire Naturelle, Paris; MPEG, Museu Paraense "Emílio Goeldi", Belém; MUCPv, Museo Universidad Nacional del Comahue, Neuquén; MZUSP, Museu de Zoologia, Universidade de Sao Paulo, Sao Paulo.

Fossil species

Postnatal ontogenetic sequences

Dinilysia patagonica (MACN-N 106, MACN-RN 976, MACN-RN 1013, MACN-RN 1014, MLP 26-410, MLP 71-VII-29-1, MPCA 527, MUCPv 38)

Adult specimens

Messelophis ermannorum (HLMD Me 7915 (Paratype), SMF ME 71, SMF ME 759, SMF ME 760, SMF ME 1370, SMF ME 1565 a+b, SMF ME 1576, SMF ME 1677, SMF ME 1805, SMF ME 1810, SMF ME 1812 a+b+c+d+e (Holotype), SMF ME

1816 a+b, SMF ME 1820, SMF ME 2608, SMF ME 11426. *Messelophis variatus* (AMNH FARB 30650, SMF ME 756, SMF ME 904, SMF ME 958 b, SMF ME 1541 a+b, SMF ME 1780 a+b, SMF ME 1805, SMF ME 1815, SMF ME 1823 (Paratype), SMF ME 1828 a+b (Holotype), SMF ME 2379 (Paratype); *Najash rionegrina* (MPCA 390-398, MPCA 385).

Extant lizard species

Postnatal ontogenetic sequences

Bachia bicolor (Tarazona et al., 2008); *Mabuya mabouya* (Jerez, 2007); *Neusticurus ecpleopus* (Bell et al., 2003); *Ophiodes intermedius* (MCN 4443, MCN 4444, FML 2304, FML 3484); *Varanus exanthematicus* (Bhullar, 2012).

Adult specimens

Feylinia currori (FML 1010, FML 1013-8); *Heloderma suspectum* (MACN 39064); *Homonota fasciata* (FML 2422); *Iguana iguana* (AMNH 1269); *Lacerta agilis* (Rieppel, 1993, 1994); *Liolaemus albiceps* (MCN 2586, MCN 2585); *Mabuya dorsivittata* (FML 896);; *Ophiodes* sp. (); *Ophisaurus apodus* (MACN 36529); *Ophisaurus* sp. (AMNH 41); *Varanus bengalensis* (AMNH 1041); *Varanus niloticus* (MLP.R 5209); *Varanus* sp. (FML 14813, FML 14814).

Extant snake species

Postnatal ontogenetic sequences

Amerotyphlops brongersmianus (MCN 4427, MCN 4428, MCN S/N); *Anilius scytale* (CENAI 3883, MACN 8817a, MACN 8817b, IB 46686, MZ 14572, MZUSP 14574); *Atractus reticulatus* (MCN 4459, MCN 4460); *Boa constrictor* (MACN 39025,

MZUSP 2553, MZUSP 13843, MCN 4432); *Bothrops diporus* (MCN 4436, MCN 4437, MCN 4438); *Epictia albipunctus* (MCN 4461, MCN 4462, MCN 4463, MCN 4464); *Hydrodynastes gigas* (MLP JW-128, MLP JW-955, MLP R-5143); *Micrurus pyrrhocryptus* (MCN 88, MCN 2189, MCN 4454, MCN 4455); *Python molurus* (AMNH 57819, AMNH 57783, AMNH 57798, AMNH 57819, AMNH 74615); *Xenodon merremi* (MACN 40015, MCN 4440, MCN 4441, MCN 4442).

Adult specimens

Achalinus formosanus (LSUMZ 19354; NHMUK 1983-192); *Acrantophis madagascariensis* (MNHN 1983.484); *Acrantophis dumerili* (MZUSP 14430); *Acrochordus granulatus* (AMNH 66367); *Acrochordus javanicus* (AMNH 140813); *Ahaetulla ahaetulla* (MNHN C 952-36/-37/-38); *Ahaetulla prasina* (MNHN C 2943-29/-30/-31); *Ahaetulla subocularis* (MNHN 1973.142A); *Antaresia childreni* (AMNH 86213); *Apostolepis dimidiata* (AMNH 62192); *Apostolepis flavotorquata* (AMNH 93559); *Aspidites melanocephala* (AMNH 18681); *Atractaspis irregularis* (MNHN 1991.4071/4072); *Bitis arietans* (CENAI 3386); *Boiruna maculata* (MACN 40006, MACN 40007); *Boiruna sertaneja* (MZUSP 7031); *Bothrops alternatus* (MACN 40018); *Bungarus fasciatus* (CENAI 3887); *Calabaria reinhardtii* (AMNH 45901, CM 147738); *Candoia aspera* (AMNH 142843); *Candoia carinata* (MZ 14111, MZ 14112); *Casarea dussumieri* (MNHN 1992-27, MNHN 1993.3382); *Causus rhombeatus* (MNHN 1991.4146/4147); *Causus maculatus* (MNHN 1991.4140); *Causus resimus* (MNHN 1991.4144); *Cerberus rhynchops* (MNHN 1991.4352); *Charina bottae* (CM 36539, MZ, 8854); *Clelia rustica* (MACN 40004); *Corallus hortulanus* (MZ 13050);; *Cylindrophis maculatus* (AMNH 85496); *Daboia russelli* (MNHN 1991.4112/4113/4114, MNHN 1997.6005/6037); *Dasypeltis scabra* (CENAI

3853); *Dendroaspis polylepis* (MACN S/N); *Enhydris bocourti* (MNHN 1970-557A); *Enhydris enhydris* (MNHN C 3458-23/-24); *Enhydris innominata* (MNHN 1970-560A); *Enhydris plumbea* (MNHN C 3461-15/-19); *Enhydris dussumieri* (MNHN 2009.0206); *Enhydris jagorii* (MNHN 1970.554A); *Enhydris sieboldii* (MNHN 2009.0204); *Epicrates angulifer* (CM 35999); *Erpeton tentaculatus* (MNHN 1970.573A); *Eryx conicus* (AMNH 89273, CM 91863); *Eryx jaculus* (MZ 14101); *Eryx johni* (AMNH 99701); *Eryx miliaris* (AMNH 143770); *Eunectes murinus* (MPEG 16443); *Eunectes notaeus* (MZUSP 7622); *Eunectes deschauensis* (MPEG 18019); *Exiliboa placata* (AMNH 102892); *Helicops leopardinus* (MACN 40014); *Homoroselaps lacteus* (MNHN 1991.4162); *Hydrophis* sp. (MNHN 1986.0596); *Langaha nasuta* (MNHN 1991.4355, MNHN 1950.178A); *Leiopython albertisii* (AMNH 95140, MZUSP 14427); *Liasis fuscus* (AMNH 86222); *Ligophis miliaris* (MACN 40013); *Ligophis poecilogyrus* (MACN 40011); *Macrelaps microlepidotus* (CENAI 3858, LSUMZ 55387); *Madagascarophis colubrinus* (MNHN C 2451-37/-38); *Malpolon monsspesulanus* (MNHN 1988.6505, MNHN 1994.4175, MNHN 1991.4358, MNHN 1991.4562); *Mastigodryas bifossatus* (MACN 40017); *Mehelya capensis* (MACN 3857); *Mimophis madagascariensis* (MNHN 1989.2917, MNHN 1989.2918, MNHN 1989.2919, MNHN 1989.2961); *Morelia viridis* (AMNH 95135, MZUSP 14428); *Morelia spilota* (MNHN 1991.4048); *Naja nivea* (CENAI 3881); *Nerodia rhombifer* (CENAI 3838); *Notechis* sp. (MNHN 1991.4100); *Oxyrhabdium modestum* (LSUMZ 11814); *Oxyrhopus rhombifer* (MACN 40010); *Pareas mollendorfi* (AMNH 27770); *Parias sumatranus* (CENAI 3783); *Philodryas patagoniensis* (MACN 40008); *Philodryas mattogrossensis* (MACN 33420); *Philotamnus hoplogaster* (CENAI 3856); *Phimophis vittatus* (MACN 40005); *Psammophis crucifer* (MNHN 1991.4214); *Psammophis lineatus* (MNHN

1989.2942); *Pseudotyphlops philippinus* (BMNH 1978.1092); *Ramphiophis togoensis* (MNHN 1991.4184); *Ramphiophis maradiensis* (MNHN 1994.0587); *Rhinophis blythi* (AMNH 85076); *Rhinophis drummondhayi* (AMNH 85076); *Sanzinia madagascariensis* (MNHN 1900.122a); *Thamnodynastes hypoconia* (MACN 40016); *Trachyboa gularis* (AMNH 28982); *Tropidophis canus* (AMNH 73066, AMNH 45839); *Tropidophis feicki* (AMNH 81128, AMNH 81132); *Tropidophis melanurus* (AMNH 82880, AMNH 46690, AMNH 93002); *Tropidophis semicinctus* (AMNH 7386); *Tropidophis tackzanowskii* (MECN 3037); *Ungaliophis continentalis* (LSUMZ 55454); *Ungaliophis panamensis* (AMNH 58845, AMNH 62639); *Uropeltis ceylonicus* (AMNH 43343); *Uropeltis pulmeyensis* (MNHN 1994-756); *Uropeltis woodmansoni* (NHMUK 1930.5.8.73–74); *Vipera ammodytes* (MNHN 1991.4068); *Vipera berus* (MNHN 1991.4128); *Vipera latasti* (MNHN 1991.4145); *Xenodon dorbignyi* (MACN 40009); *Xylophis perroteti* (MNHN 1991.4426).

Three-dimentional reconstructions based on HRXCT data

Comparative specimens available from the Digital Morphology Library at the University of Texas (www.digimorph.com)

Angolosaurus skoogi (juvenile) California Academy of Sciences (CAS 206977)

Angolosaurus skoogi (adult) California Academy of Sciences (CAS 206978)

Anilius scytale (adult) National Museum of Natural History (USNM 204078)

Bothrops asper (adult) Field Museum of Natural History (FMNH 31162)

Casarea dussumieri (adult) University of Michigan Museum of Zoology (UMMZ 190285)

Chilabothrus striatus (adult) National Museum of Natural History (USNM 59918)

Heloderma suspectum (juvenile) Texas Memorial Museum (TNHC 62767)

Heloderma suspectum (adult) Texas Memorial Museum (TNHC 62766)

Lampropeltis getula (late embryo) Texas Memorial Museum (uncatalogued)

Lampropeltis getula (adult) Field Museum of Natural History (FMNH 95184)

Loxocemus bicolor (adult) Field Museum of Natural History (FMNH 104800)

Micrurus fulvius (adult) Field Museum of Natural History (FMNH 39479)

Shinisaurus crocodylurus (juvenile) Texas Memorial Museum (TNHC 62987)

Shinisaurus crocodylurus (adult) Field Museum of Natural History (FMNH 215541)

Trimorphodon biscutatus (adult) Field Museum of Natural History (FMNH 42171)

Typhlops jamaicensis (adult) National Museum of Natural History (USNM 12378)

Varanus gouldi (adult) Texas Memorial Museum (TMM M-1295)

Xenopeltis unicolor (adult) Field Museum of Natural History (FMNH 148900)

B. Quantitative analysis

	svl	scl	scl/av scl	pml	ql	sl	ml
Ophiodes intermedius	32	6.7	0.59	4.8	1.3	0.8	5.1
Ophiodes intermedius	56	8.1	0.71	6.8	1.6	1.1	7.6
Ophiodes intermedius	114	11.8	1.04	9.8	2	1.7	10.9
Ophiodes intermedius	237	18.9	1.66	16.5	3.2	2.8	17.8
Epictia albipunctus	85	4.2	1.12	2.4	1.6	n	1.6
Epictia albipunctus	145	4.7	1.25	3	1.7	n	1.8
Epictia albipunctus	218	4.8	1.28	3	1.6	n	1.9
Epictia albipunctus	276	5.1	1.36	3.1	1.8	n	2.1
Anilius scytale	665	16.7	0.64	16.1	2.5	1.9	16.5
Anilius scytale	?	18.7	0.72	16.6	3	2.7	17.4
Anilius scytale	?	25.9	0.99	23.7	3.3	3.1	24.9
Anilius scytale	895	27.1	1.04	24.9	3.7	3.3	25.4
Anilius scytale	?	42.1	1.61	40.9	6.1	5.8	39.2
Boa constrictor	311	20.5	0.43	18.7	5.8	4.3	21.1
Boa constrictor	463	24.4	0.52	28.5	8.8	7	25
Boa constrictor	1623	50.2	1.06	58.8	13.8	17.9	59.2
Boa constrictor	?	68.3	1.45	78.7	19	23.9	79.9
Boa constrictor	3124	72.4	1.53	80.2	20.2	25.6	81.1
Micrurus pyrrhocryptus	182	8.3	0.57	6.2	1.2	1.1	7.5
Micrurus pyrrhocryptus	395	12.1	0.84	10.8	2	2.5	12.5

Micrurus pyrrhocryptus	802	17.8	1.23	15.2	3.5	3.7	18.6
Micrurus pyrrhocryptus	921	19.5	1.35	18.3	4.2	4.5	21.2
Bothrops diporus	229	13.6	0.67	14.3	4.6	3.9	16.2
Bothrops diporus	509	17.5	0.86	19.8	7.2	4.8	22.1
Bothrops diporus	554	18.9	0.93	22.7	8.2	5.7	24.6
Bothrops diporus	762	25.8	1.26	30.7	10.6	7.8	33.1
Bothrops diporus	892	26.3	1.29	33.4	13.5	9.1	37.5
Xenodon merremi	157	11.2	0.57	12.8	3.9	3.4	12.8
Xenodon merremi	202	12.6	0.64	14.2	5.5	4.1	15.3
Xenodon merremi	492	19.9	1.01	23.8	10	7.8	25.2
Xenodon merremi	753	27.1	1.37	34.2	15.4	12.5	37.6
Xenodon merremi	870	27.9	1.41	35	16.8	11.6	38.2
Python molurus	?	24.1	0.3	23.4	4.9	6.3	25.2
Python molurus	?	54.8	0.68	60	13.6	18.1	61.1
Python molurus	?	78.8	0.97	85.9	23.3	25.4	90.6
Python molurus	?	104.3	1.29	123.4	34.8	39	125.2
Python molurus	?	142.1	1.76	168.9	39	43.8	147.7

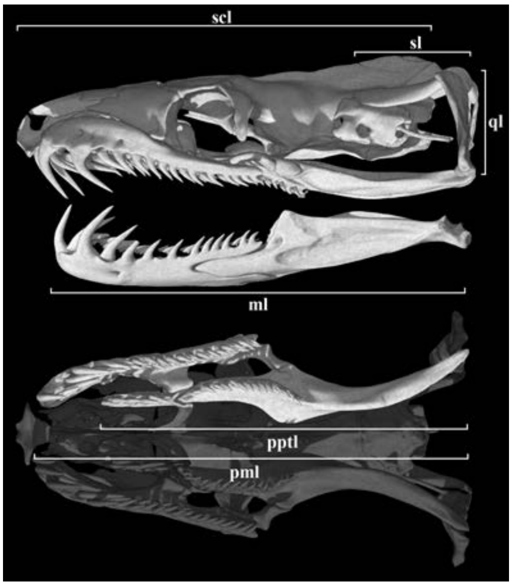

Selected measurements (in mm). Due to the large variation in snout-condyle length (hereafter, SCL), I rescaled the value of the SCL for each species dividing it with the average of the SCL of all specimens of each species. ml, mandible length; pml, palatomaxillary bar length; pptl, pterygopallatine bar length; ql, quadrate length; scl, snout-condyle length; sl, supratemporal length; svl, snout-vent length.

C. Macrohabitat definitions

For several years, scientists have employed different terms to define macrohabitats occupied by snakes, most of which have not been matched by the development of explicit definitions. Moreover, in much of the literature authors have coined several terms in order to describe intermediate macrohabitats (e.g. semifossorial, semiaquatic) that introduced more confusion. Regrettably, this situation entails the negative consequence of hinder comparisons between macrohabitats and their evolutionary implications for species that occupy them. Hence, I provide an explicit terminology that imparts each macrohabitat with information about its anatomical correlates in snakes. However, a caveat must be made because some snakes are capable to occupy different macrohabitats without specific anatomical structures. A good example of this situation is the arboreal capabilities of several snakes, even present in fossorial scolecophidians of the family Leptotyphlopidae (Vanzolini, 1970; pers. obs.). This attempt is based on previous works that employed macrohabitat definitions, in special the papers of Duellman (1978), Cadle and Greene (1993) and Martins and Oliveira (1998).

Underground macrohabitats

It is important to note that most snakes are facultatively sheltering due to the ecological plasticity of their elongated limbless bauplan. Despite of this plastic condition, some groups display habitat specializations, which usually are correlated with discrete anatomical traits. Most basal groups of snakes and some groups of macrostomatans inhabit in tunnels, crevices or below of different strata composed by many kinds of leaf litters and other loose substrates present in poorly consolidated substrates. Common among snakes that occupy underground macrohabitats is the small body size (SVL < 1000 mm), strong to moderate reduction of eyes and consequently orbits, reduction of the number of dorsal scale rows, fusion of head scales and strong abbreviation of the tail (Bogert, 1947; Inger and Marx, 1965; Downs, 1967; Gans, 1974; Shine and Wall, 2008). Because head is the principal locomotor organ in this kind of snakes, head morphology is strongly constrained due to the ability to penetrate the substrate and exposure of cranial bones to stress. Head width also may be reduced and cranial rigidity increased by shortening the supratemporals and quadrates whit the subsequent shortening of the lower jaws, robustness and shape modification of the premaxilla bone and naso-frontal joint, narrowing of the braincase and rearrangement of muscular and glandular elements (Gans, 1974; Savitzky, 1978, 1983). Associated with the reduction of body diameter, neural spines are strongly reduced or absent (Johnson, 1955).

Fossorial (=burrowing, subterranean, active burrower)

Fossorial snakes are those capable of burrowing and/or using soil galleries and regularly spend their activity time in the soil. I consider truly fossorial snakes all

scolecophidians and basal alethinophidian such as uropeltids and species of the genus *Anomochilus*. These snakes exhibit strong body modifications such as rigid skull and very narrow heads (usually both body extremes are similar in shape), strongly reduced eyes covered by a scale and modifications in the caudal scalation to form tail shields or spines. Nearly all species of these groups have thin, small bodies (SVL < 600 mm), and a homogeneous cylindrical body shape.

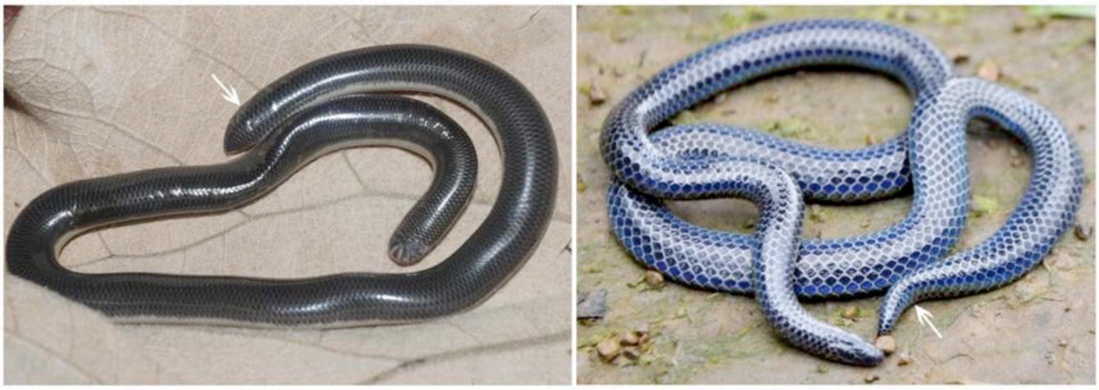

Representatives of fossorial snakes. Left, the typhlopid *Argyrophis muelleri* (SVL 550 mm) (Credit: www.indiansnakes.org); right, the uropeltid *Melanophidium wynaudense* (SVL 300 mm) (Credit: https://bangkokherps.wordpress.com). White arrow indicates the position of the vent.

Cryptozoic (=sub fossorial, sand swimmers, crevice-dwellers, leaf litter inhabitants, litter-swimmers)

Are those snakes that regularly spend part of their activity time inside the leaf litter or other kinds of loose substrate. Usually, these snakes exhibit slightly slender body sizes than fossorial forms. We consider truly cryptozoic snakes basal alethinophidians such as *Anilius scytale* and species of the genus *Cylindrophis*, basal macrostomatans *Xenopeltis* (*X. unicolor* and *X. hainanensis*) and *Loxocemus bicolor*, erycine boas, and some species of caenophidians. Within this last clade exist several cryptozoic forms, some of which represent entire subclades such as elapomorphine

dipsadids, old and new world coral snakes, african atractaspidids, and sonorine colubrines, among others.

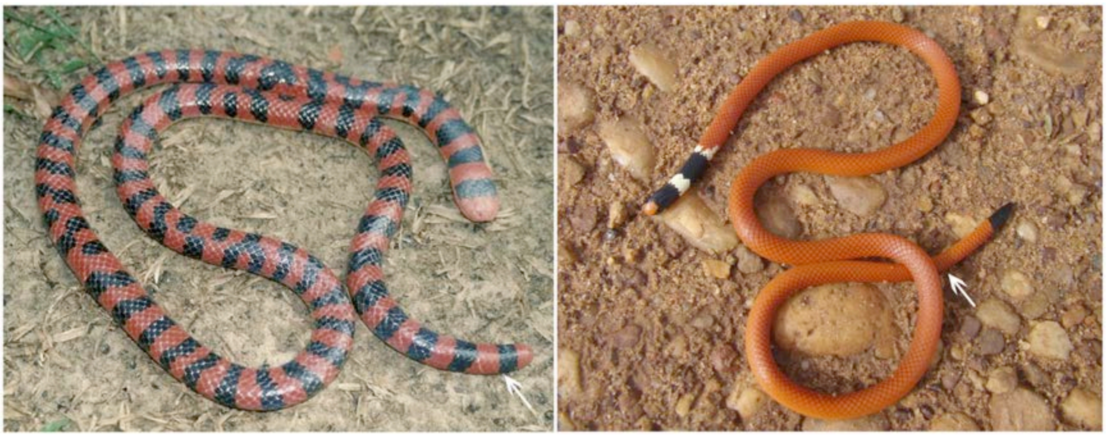

Representatives of cryptozoic snakes. Left, the basal alethinophidian *Anilius scytale* (SVL 1100 mm); right, the colubroid *Apostolepis ammodites* (SVL 500 mm) (Credit: www.licenciamento.ibama.gov.br). White arrow indicates the position of the vent.

Surface macrohabitats

Snakes that occupy surface macrohabitats display an important range of body sizes (SVL ≈ or > 1000 mm) including large forms that can reach several meters. Surface snakes spend most part of their activity time above the substrate, climbing plants or in the water column. Due to the relevance of the postcranial body as locomotor organ in surface snakes, exist some anatomical traits related with the different surface macrohabitats. Related with the increase of body size, surface snakes have more vertebrae than underground snakes (Lindell, 1994). It is important to note that few taxa exhibit characters present in underground forms. An example of this is the shortening of the suspensorium and jaws present in presumably paedomorphic surface snakes such as some boine species of the genus *Chilabothrus* (Kluge, 1989).

Terrestrial (= ground inhabitants, surface-dwelling)

Terrestrial snakes are those that regularly spend this activity time on the ground, above to different kind of substrates (leaf litter, sand, rocks, etc). Terrestrial snakes display the most generalised forms with respect of body shape. It is important to underline that largest snake species (SVL > 3000 mm) occupy this macrohabitat.

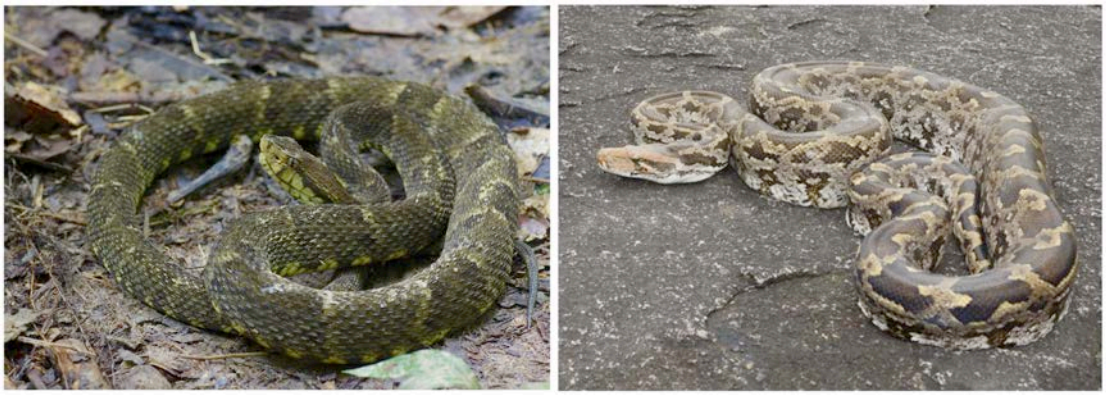

Representatives of terrestrial snakes. Left, the colubroid *Bothrops atrox* (SVL 1600 mm) (Credit: www.jungledragon.com); right, the booid *Python molurus* (SVL 700 mm) (Credit: www.novanature.org).

Arboreal (=bush and tree inhabitants)

Arboreal snakes are those that regularly spend part of their activity time on the vegetation (epiphytes, vines, shrubs and trees) above the ground level (Martins and Oliveira, 1999). Their body is usually slender and thin, and usually tail is more slender than terrestrial or aquatic forms (Marx and Rabb, 1972; Lillywhite and Henderson, 1993; Lindell, 1994). Usually, arboreal forms have more precloacal vertebrae than terrestrial and aquatic forms, and some species of colubroids (e.g. *Leptophis ahaetula*) have elongated vertebral centra.

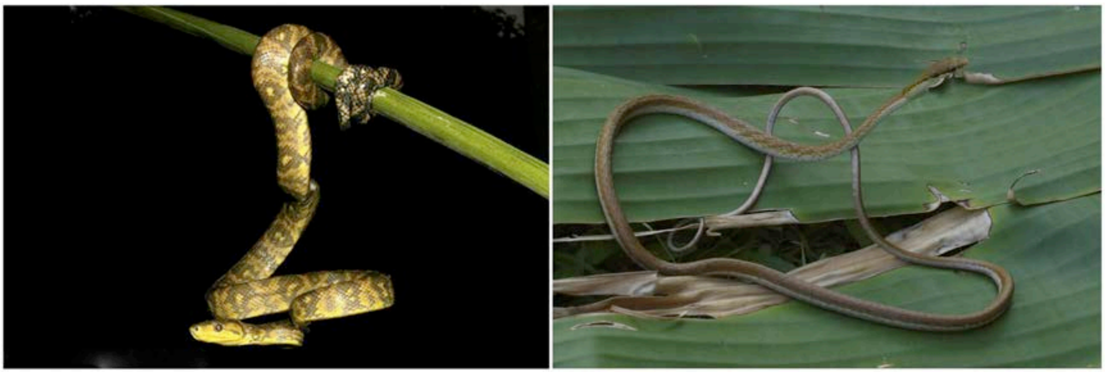

Representatives of arboreal snakes. Left, *Corallus ruschenbergerii* (SVL 1500 mm) (Credit: www.guaquira.net); right, *Leptophis stimsoni* (SVL 700 mm) (Credit: www.flickr.com).

Aquatic (=water snakes, marine snakes)

Aquatic indicates those snakes that regularly spend part of their activity time inside the water. Some freshwater forms like the boid *Eunectes*, homalopsine and hydropsine colubroids show external nares and eyes in the dorsal region of the head. Sea snakes exhibit a dorso-ventrally elongated body (due to a prominent ventral keel), and the tail (a major propulsive structure during swimming) is higher and thinner than in terrestrial snakes (i.e., paddle-shaped) but shorter relative to body length (Brischoux and Shine, 2011). Simoliophiids were fossil Mesozoic snakes recovered invariably in marine sedimentary formations, which display some particular postcranial traits usually attributed to a marine habitat such as pronounced pachyostosis in vertebrae and ribs (Lee and Caldwell, 1998; Rieppel et al., 2003; Rage and Escuillié, 2000), and elongated haemal arches in postcloacal region (but see Rieppel and Head [2006] for the homology status of these caudal structures).

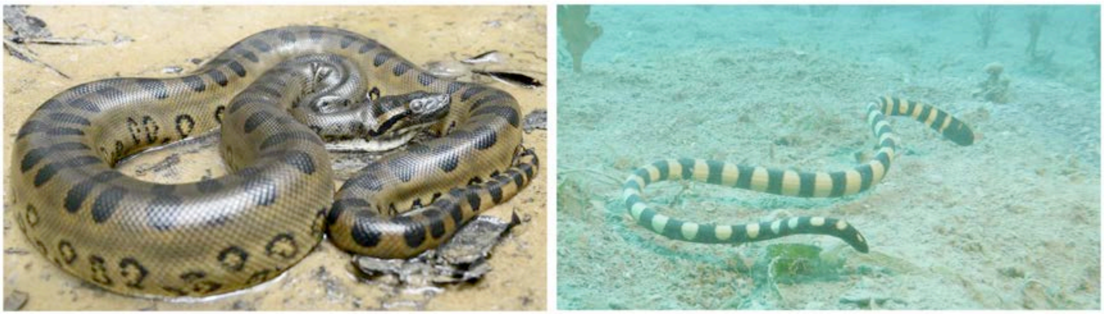

Representatives of aquatic snakes. Left, *Eunectes murinus* (SVL 6000 mm) (Credit: https://respectgreen.wordpress.com); right, *Hydrophis coggeri* (SVL 1350 mm) (Credit: www.endemia.nc).

D. Prey types

This definition of prey types is reproduced with some modifications from the classical work of Cundall and Greene (2000) about snake feeding.

Type I

Preys of this type consist on relatively small items in mass and cross-sectional area regardless of shape, such as adult insects and their different developmental stages (pupae, larvae), fish eggs, among others.

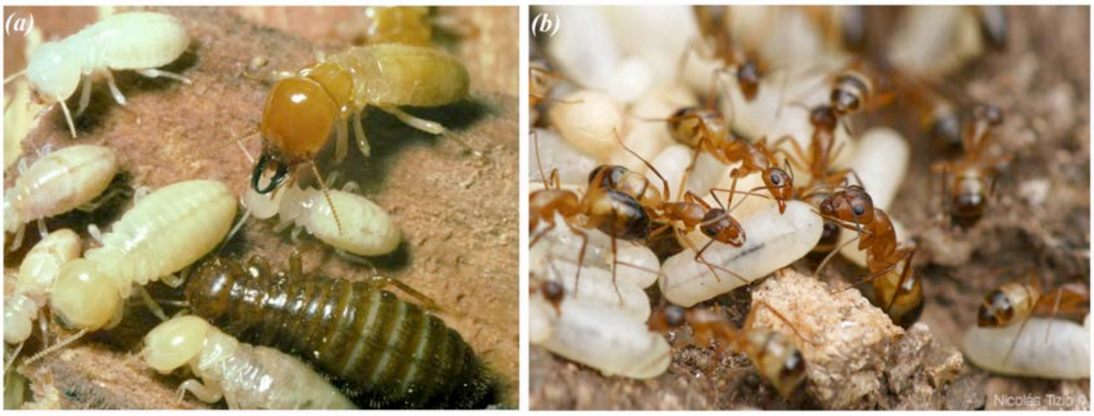

Representatives of Type I preys. (a) nymphs and adults of termite *Mastotermes darwiniensis* (weight: 0,004 g; length: 3-5 mm) (Credit: http://darwinblog. blogspot.com.ar/); (b) pupae and adults of ants of the family Formicidae (weight: 0,005 g; length: 5-8 mm) (Credit: www.biodiversidadvirtual.org).

Type II

Type II preys are elongate, often without protruding locomotor organs like arthropod appendages or tetrapod limbs. I include in this category earthworms, elongated fishes, legless tetrapods, small elongated legged lizards and soft-shelled squamate eggs. This kind of preys has a small cross-sectional area.

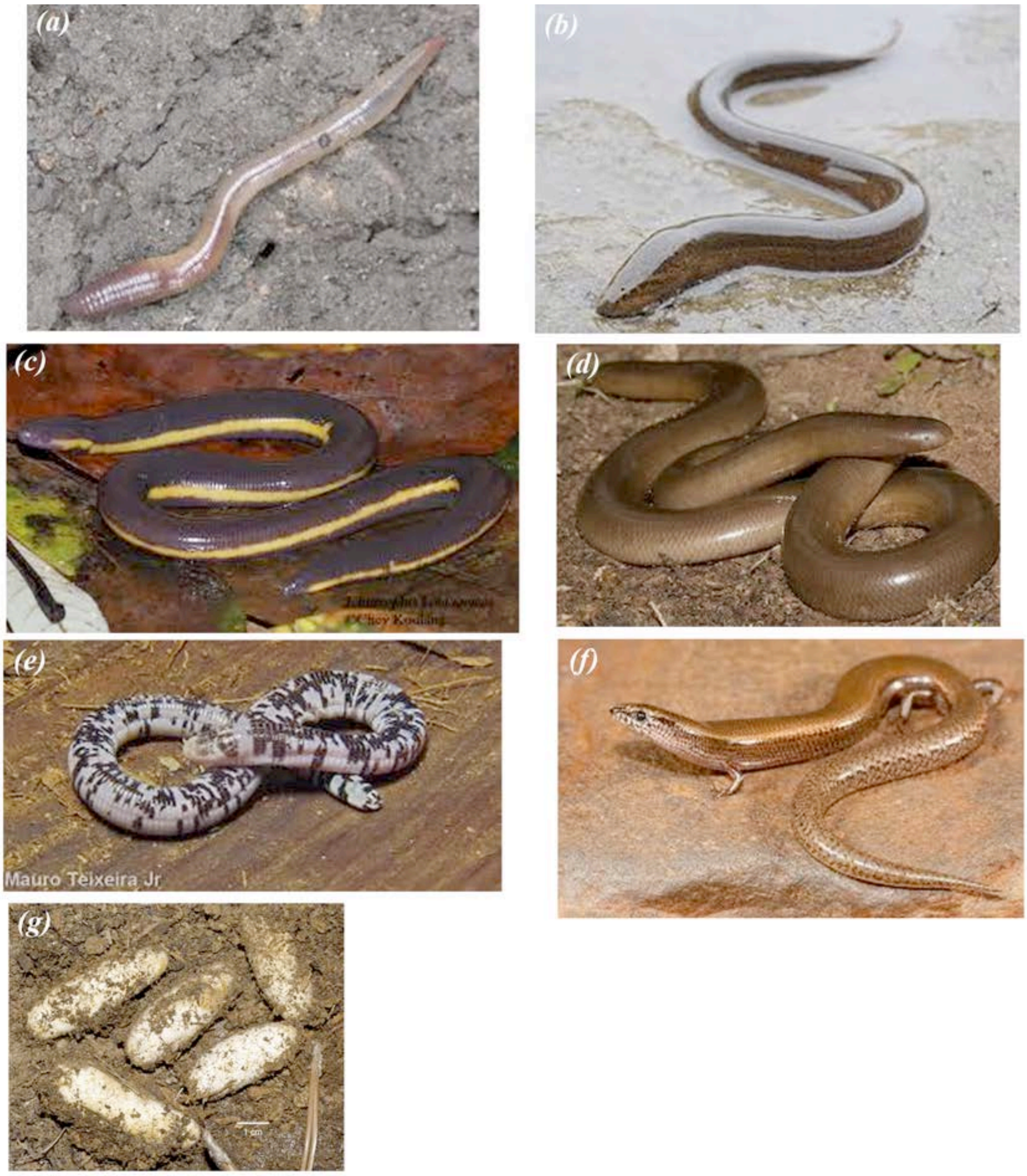

Representatives of Type II preys. (a) Earth worm *Lumbricus* sp. (weight: 0,6 g; length: 50 mm) (Credit: www.teara.govt.nz); (b) the Asian swamp eel *Monopterus albus* (weight: 10 g; length: 240 mm) (Credit: http://safeimufti. wordpress.com); (c) the caecilian *Ichthyophis kohtaoensis* (weight: 34 g; length: 343 mm) (Credit:); (d) spotted worm lizard *Amphisbaena fuliginosa* (weight: 10 g; length: 190 mm) (Credit: http:// calphotos.berkeley.edu); (e) South African blind snake *Afrotyphlops bibronii* (weight: 34 g; length: 320 mm) (Credit: www.sareptiles. co.za/forum/viewtopic.php?f=141&t=26352&start=120); (f) Australian skink *Lerista muelleri* (weight: 4 g; length: 100 mm) (Credit: www.arod.com.au); (g) eggs of colubrid snake *Coluber constrictor* (weight: 2 g; length 50 mm) (Credit: http://www. susanleachsnyder.com/GopherTortoise Preserve/Reptiles.html).

Type III

Type III prey are fusiform or roughly spherical as well as relatively heavy so they necessitate compensation for both high handling cost and increased gape. We incorporate large lizards in this category.

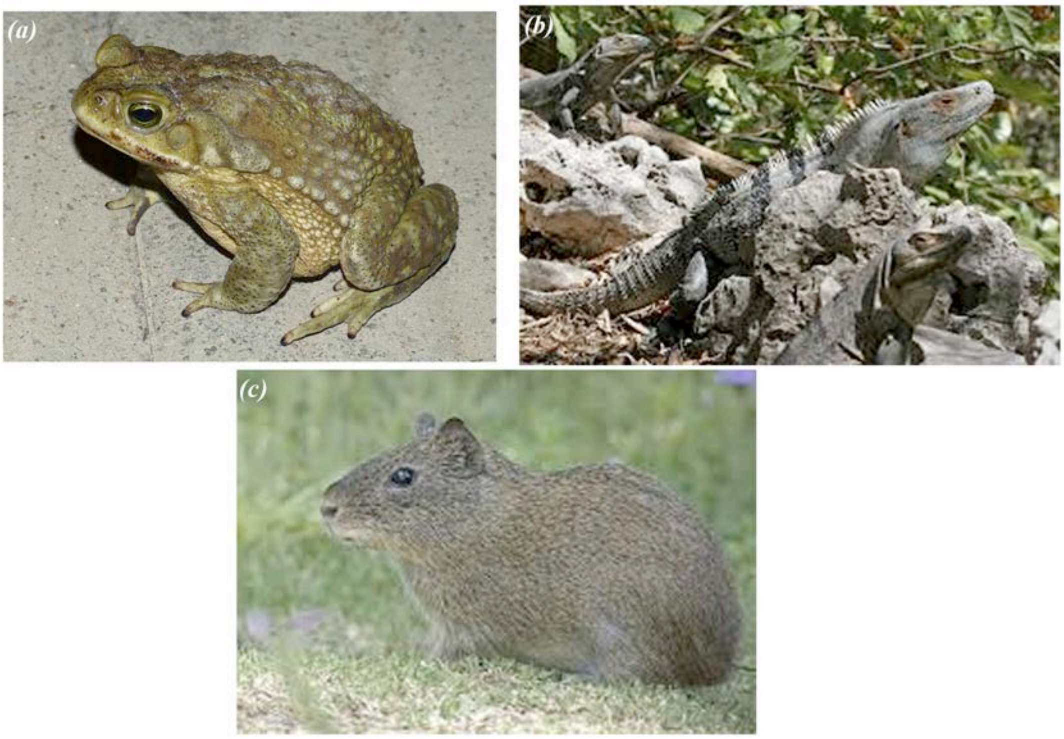

Representatives of Type III preys. (a) toad *Rhinella arenarum* (weight: 130 g; length: 100 mm) (Credit: http://amiralles.com); (b) black iguana *Ctenosaura similis* (weight: 300 g; length: 540 mm) (Credit: http://en.wikipedia.org); (c) guinea pig *Cavia aperea* (weight: 350 g; length: 200 mm) (Credit: http://elaniorapaz.blogspot.com).

Type IV

Type IV prey weigh substantially less than predicted by their maximum diameter, either because their cross-sectional shape is not circular (e.g., many fishes) or they possess protruding body parts (e.g. wings of birds and bats) so they require large gape but not specialized immobilization mechanisms.

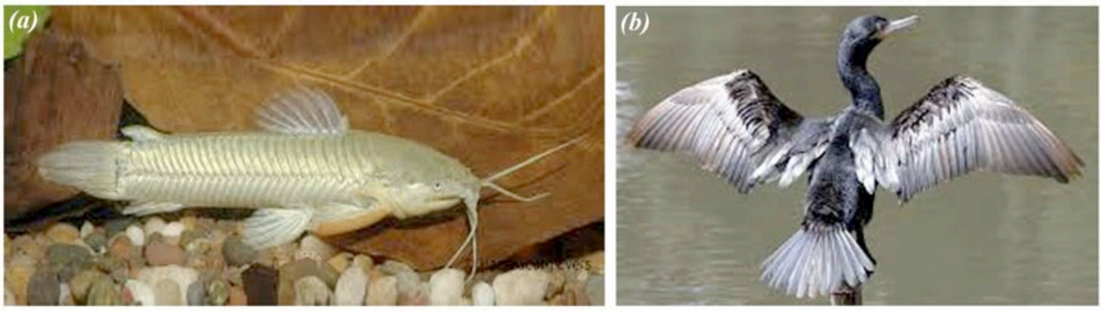

Representatives of Type VI preys. (a) catfish *Callichthys callichthys* (weight: 80 g; length: 170 mm) (Credit: www.planetcatfish.com); (b) neotropical cormorant *Phalacrocorax brasilianus* (weight: 1000 g; length: 640 mm) (Credit: www.ebirdr.com).

E. Phylogenetic MANOVA

Type I (Sequential) Sums of Squares and Cross-products
Randomization of Raw Values used

	df	SS	MS	Rsq	F	Z	P.value
Size	1	246.8	246.764	0.062554	8.274	4.521	0.0042996
Residuals	124	3698.0	29.823				
Total	125	3944.8					

Results of the phylogenetic MANOVA performed in R used to compare the significance of differences between most of the species employed in the morphological-ecological survey for the macrohabitat and prey type.

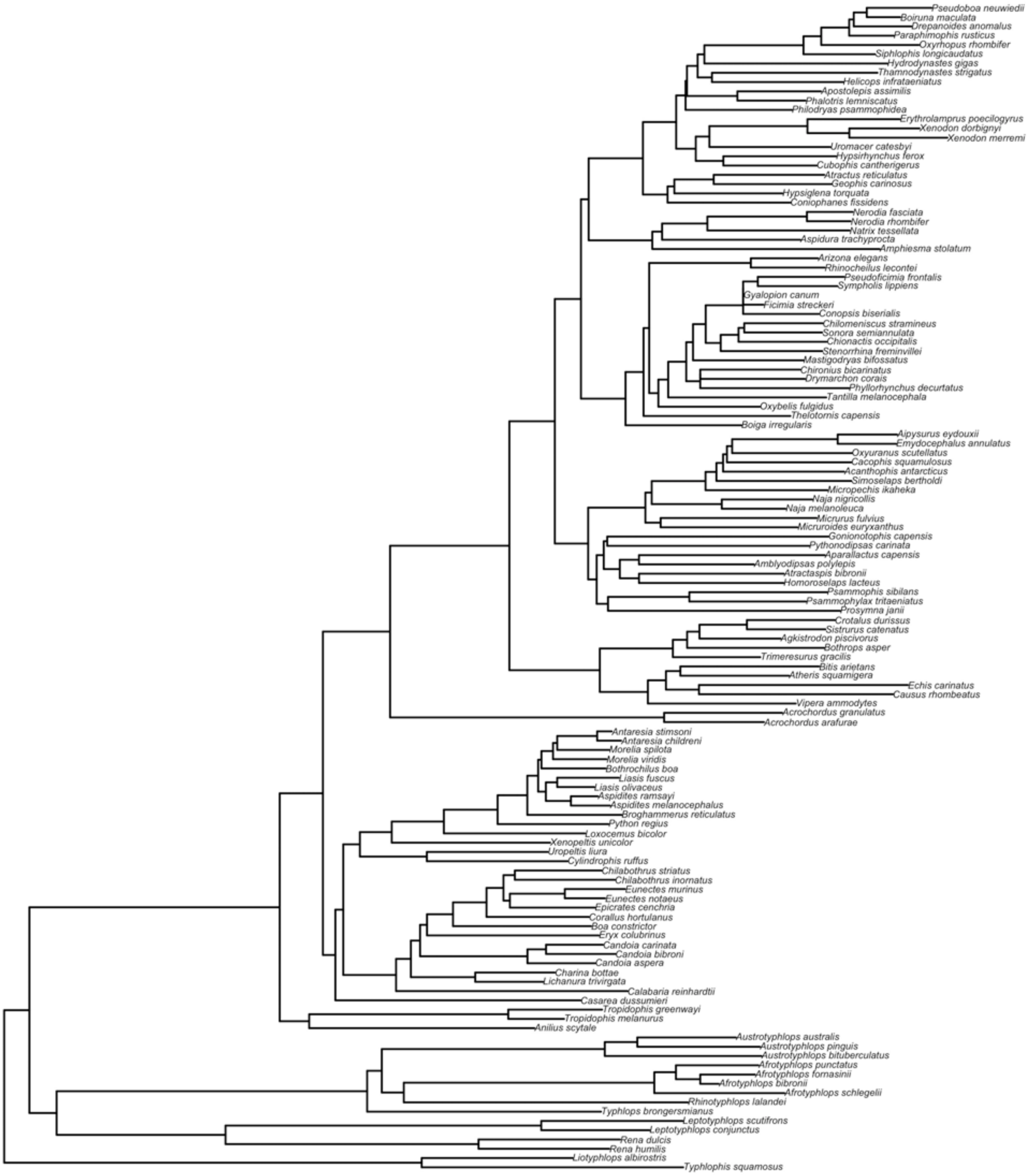

Phylogenetic tree utilized in the MANOVA analysis. This tree corresponds with the maximum-likelihood tree published by Pyron et al. (2013), which was pruned to include only the species employed in this study.

F. Supplementary figures

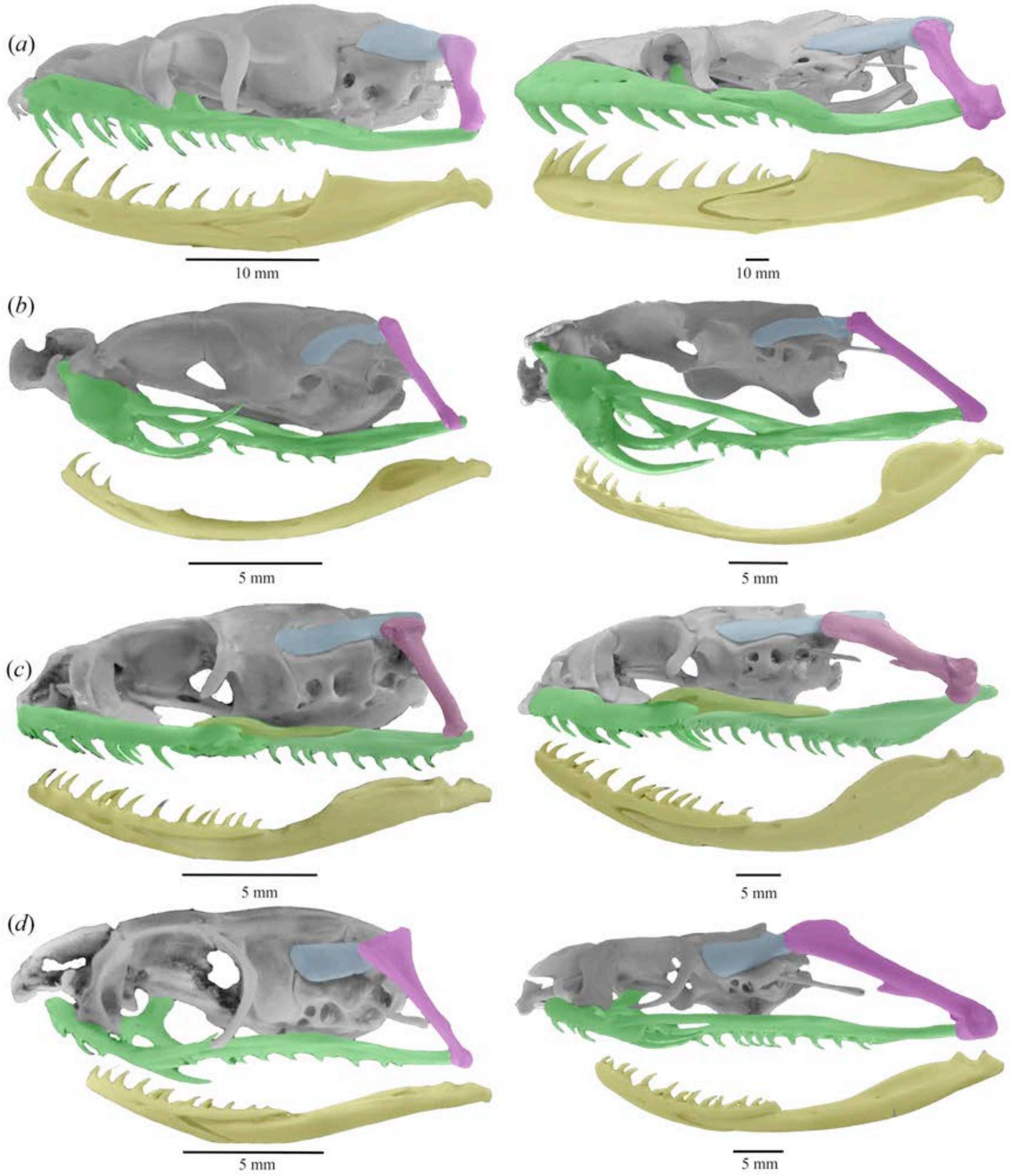

Figure S1. A, lateral view of the skull of a juvenile (SVL 489 mm; left) and adult (SVL 4030 mm; right) specimens of the pythonid *Malayopython reticulatus*; B, juvenile (SVL 251 mm; left) and adult (SVL 848 mm; right) specimens of the viperid *Bothrops diporus*; C, juvenile (SVL 289 mm; left) and adult (SVL 1108 mm; right) specimens of the dipsadid colubroid *Hydrodynastes gigas*; D, juvenile (SVL 231 mm; left) and adult (SVL 990 mm; right) specimens of the dipsadid colubroid *Xenodon merremi*.

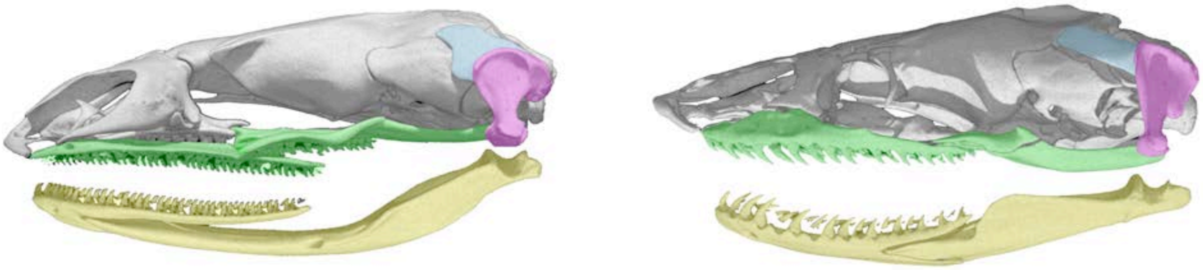

Figure S2. Three-dimensional reconstruction of the lateral view of the skull of *Xenopeltis unicolor* (left) and *Loxocemus bicolor* (right) based on HRXCT data. Not to scale.

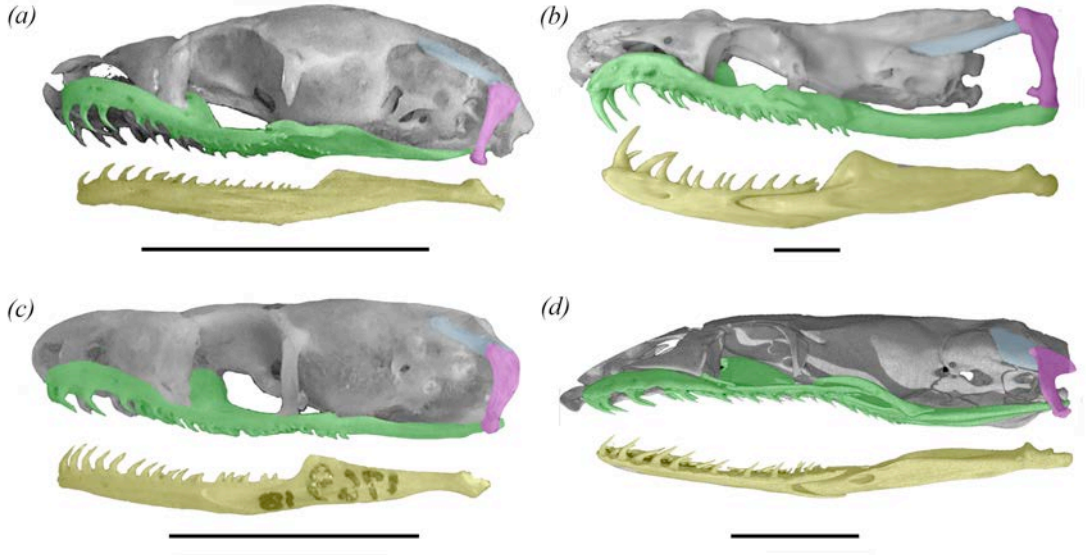

Figure S3. Lateral view of the skull of a juvenile (SVL 390 mm; a) and adult (SVL 2030 mm; b) specimens of *Chilabothrus subflavus*, adult specimen of *Chilabothrus gracilis* (SVL 760 mm; c), and adult specimen of *Casarea dussumieri* (three-dimensional reconstruction based on HRXCT data). Scale bar 5 mm.

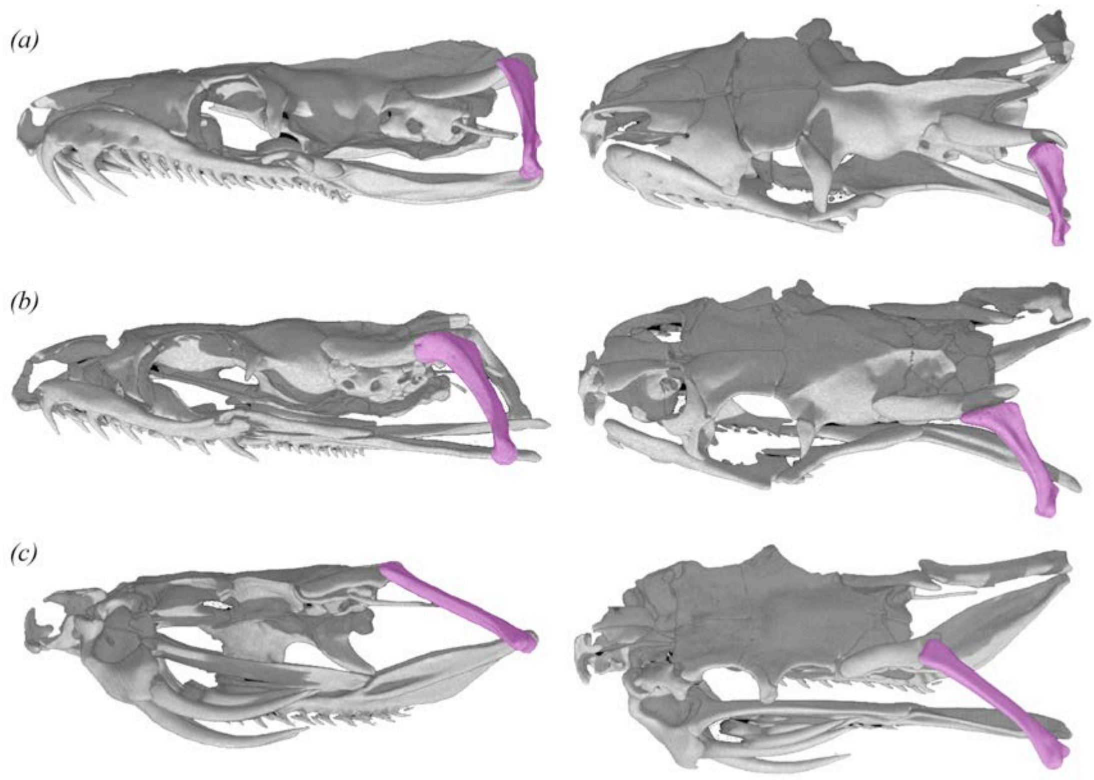

Figure S4. Three-dimensional reconstructions of the skull in lateral (left) and dorsolateral (right) views of adult specimens of the boine *Chilabothrus striatus* (a), the colubrid *Trimorphodon biscutatus* (b), and the viperid *Bothrops asper* based on HRXCT data. Not to scale.

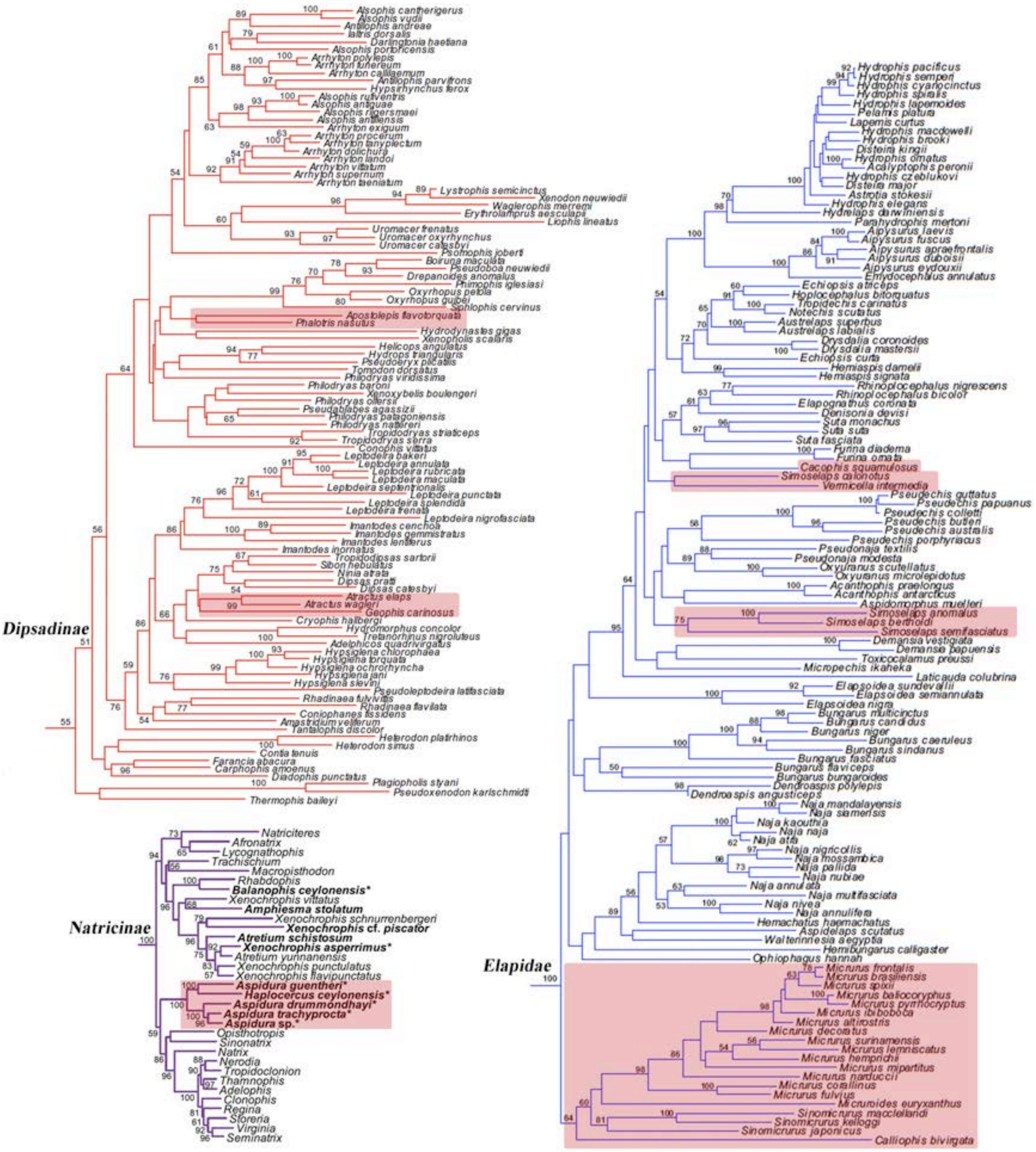

Figure S5. Molecular phylogeny of colubroid snakes (Pyron et al., 2011, 2013). Red rectangles highlight cryptozoic species/clades.

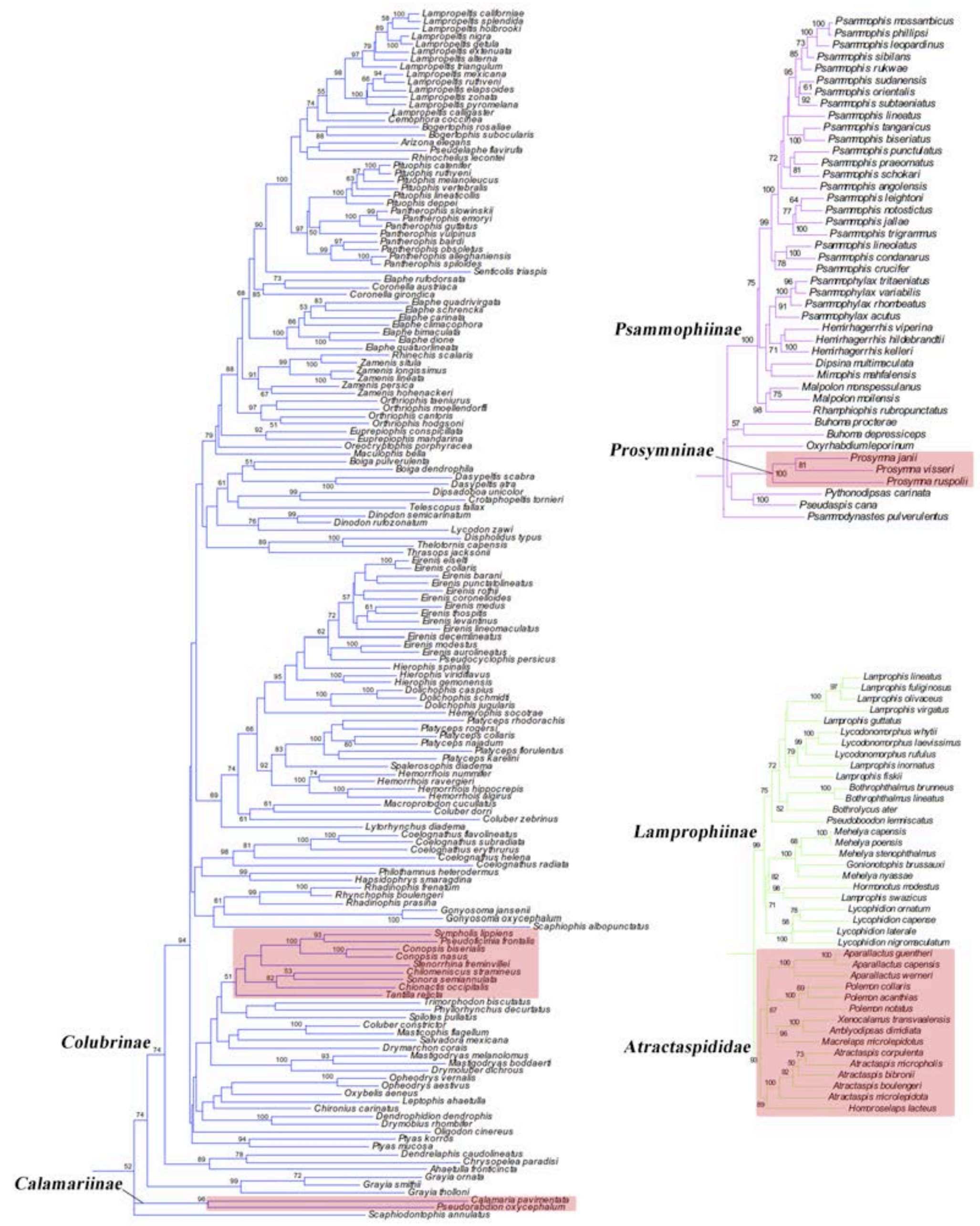

Figure S5 (continued).

F. References for electronic supporting materials

Aguiar LFS, Di-Bernardo M. 2004 Diet and feeding behavior of *Helicops infrataeniatus* (Serpentes: Colubridae: Xenodontinae) in southern Brazil. *Stud. Neotrop. Fauna Environ.* **39 (1)**, 7-14.

Akani GC, Angelici FM, Luiselli L. 2005 Ecological data on the Goldie's tree cobra, *Pseudohaje goldii* (Elapidae) in southern Nigeria. *Amphibia-Reptilia* **26 (3)**, 382-387.

Akani GC, Luiselli L, Eniang EA, Amuzie CC, Ebere N. 2007 Aspects of the ecology of the spotted blindsnake, *Typhlops punctatus punctatus* in Port-Harcourt, Nigeria. *Afr. J. Ecol.* **46**, 533-539.

Alencar LRV, Gaiarsa MP, Martins M. 2013 The evolution of diet and microhabitat use in Pseudoboine snakes. *South. Am. J. Herpetol.* **8**, 60-66.

Andrade RO, Silvano RAM. 1996 Comportamento alimentar e dieta da falsa-coral *Oxyrhopus guibei* Hoge & Romano (Serpentes, Colubridae). *Rev. Bras. Zool.* **13 (1)**, 143-150.

Antunes A, Haddad CFP. 2009 *Tropidophis paucisquamis* (Brazilian Dwarf Boa). Diet and caudal luring. *Herp. Rev.* **40 (1)**, 104.

Avila RW, Ferreira VL, Souza VB. 2006 Biology of the blindsnake *Typhlops brongersmianus* (Typhlopidae) in a semideciduous forest from central Brazil. *Herpetol. J.* **16**, 403-405.

Avila RW, Kawashita-Ribeiro RA, Ferreira VL, Strüssmann C. 2010 Natural history of the oral Snake *Micrurus pyrrhocryptus* Cope 1862 (Elapidae) from semideciduous Forests of Western Brazil. *South Am. J. Herpetol.* **5 (2)**, 97-101.

Babb RD, Bradley GL, Brennan TC, Holycross AT. 2005 A preliminary assessment

of the diet of *Gyalopion quadrangulare* (Serpentes: Colubridae). *Southwest. Nat.* **50 (3)**, 390-392.

Barlow A, Pook CE, Harrison RA, Wüster W. 2009 Co-evolution of diet and prey-specific venom activity supports the role of selection in snake venom evolution. *P. R. Soc. B* **276**, 2443-2449.

Baruah M, Das M, Sengupta S. 2001 Food and feeding of *Amphiesma stolatum* (Linnaeus 1758). *J. Environ. Biol.* **22(4)**, 315-317.

Bell CJ, Evans SE, Maisano JA. 2003 The skull of the gymnophthalmid lizard *Neusticurus ecpleopus* (Reptilia: Squamata). *Zool J Linn Soc* **139**, 283-304.

Bernarde PS, Macedo LC. 2006 *Phalotris matogrossensis* (False Coral Snake). Diet. *Herp. Rev.* **37 (2)**, 234.

Bhullar B-AS. 2012 A phylogenetic approach to ontogeny and heterochrony in the fossil record: cranial evolution and development in anguimorphan lizards (reptilia: squamata). *J Exp Zool Part B* **318**, 521-530.

Boback SM. 2005 Natural history and conservation of island boas (*Boa constrictor*) in Belize. *Copeia* **2005**, 880-885.

Bogert CM. 1947 The status of the genus *Leptodrymus* Amaral, with comments on modifications of colubrid pre-maxillae. *Am. Mus. Nov.* **1352**, 1-14

Boughner JC, Buchtova M, Fu K, Diewert VW, Hallgrımsson B, Richman JM. 2007 Embryonic development of *Python sebae*. I: staging criteria and macroscopic skeletal morphogenesis of the head and limbs. *Zoology* **110**, 212-230.

Branch WR, Shine R, Harlow PS, Webb JK. 1997 Sexual dimorphism, diet and aspects of reproduction of the western keeled snake, *Pythonodipsas carinata* (Serpentes: Colubridae). *J. Herpetol. Assoc. Afr.* **46**, 89-97.

Braña F, Bea A, Saint Girons H. 1988 Composición de la dieta y ciclos de

alimentación en *Vipera seoanei* Lataste, 1879. Variaciones en relación con la edad y el ciclo reproductor. *Munibe* **40**, 19-27.

Brattstrom BH, Schwenkmeyer RC. 1951 Notes on the natural history of the worm snake, *Leptotyphlops humilis*. *Herpetologica* **7**, 193-196.

Braz HB, Kasperoviczus KN, Almeida-Santos SM. 2014 Reproductive ecology and diet of the fossorial snake *Phalotris lativittatus* in the Brazilian Cerrado. *Herpetol. J.* **24(1)**, 49-57.

Brischoux F, Shine R. 2011. Morphological adaptations to marine life in snakes. *J. Morphol.* **272 (5)**, 566-572.

Broadley DG. 1958 Feeding habits of *Calamelaps* and *Aparallactus*, *J. Herpetol. Assoc. Rhodesia* **3**, 7.

Broadley DG. 1979 Predation on Reptile Eggs by African Snakes of the Genus *Prosymna*. *Herpetologica* **35 (4)**, 338-341

Brongersma LD. 1934 Contributions to Indo-Australian Herpetology. *Zool. Meded.* **17**, 161-251.

Bullock DJ. 1986 The ecology and conservation of reptiles on Round island and Gunners Quoin, Mauritius. *Biol. Conserv.* **37 (2)**, 135-156.

Bullock DJ, North S, Alexander JB. 1979 Report of the 1975 and 1978 Edinburgh University expeditions to Round Island (Mauritius). Section 11 (Reptiles). Unpublished report, Edinburgh University Library.

Cadle JE, Greene HW. 1993 Phylogenetic patterns, biogeography, and the ecological structure of neo-tropical snake assemblages. In *Species diversity in ecological communities: historical and geographic perspectives* (eds RE Ricklefs, D Schluter), pp. 281-293. Chicago, USA: University of Chicago Press.

Campbell JA, Ford LS, Karges JP. 1983 Resurrection of *Geophis anocularis* Dunn with comments on its relationships and natural history. *Trans. Kansas Acad. Sci.* **86**, 38-47.

Castañeda-Gonzalez O, Manjarrez J, Goyenechea I, Fajardo V. 2011 Ecology of a population of the earthsnake *Conopsis biserialis* in the mexican transvolcanic axis. *Herpetol. Conserv. Biol.* **6 (3)**, 364-371.

Céspedez JA, Lamas MF, Ruiz García JA. 2014 Dieta de la culebra *Xenodon merremi* (Squamata: Dipsadidae) en una localidad del chaco húmedo de Argentina. *Bol. Mus. Nac. Hist. Nat. Paraguay* **18 (1)**, 104-107.

Cisneros-Heredia DF. 2005 Report of molluscivory in *Atractus carrioni. Herpetozoa* **18**, 185-186.

Cobb VA. 2004 Diet and Prey Size of the Flathead Snake, *Tantilla gracilis*. *Copeia* **2004 (2)**, 397-402.

Cundall D, Greene HW. 2000 Feeding in snakes. In *Feeding: Form, Function and Evolution in Tetrapod Vertebrates* (ed K Schwenk), pp. 293–333. San Diego, USA: Academic Press.

Cundall, D. & Irish, F. 2008. The snake skull. In *Biology of the Reptilia*, vol. 20, The skull of Lepidosauria (eds. C. Gans, A. S. Gaunt & K. Adler), pp. 349-692. Ithaca: New York Society for the Study of Amphibians and Reptiles.

Cundall, D., Wallach, W. & Rossman, D. A. 1993. The systematic relationships of the snake genus *Anomochilus*. *Zool. J. Linn. Soc.* **109**, 275-299.

Cunha OR, Nascimento FP. 1978 Ofídios da Amazônia X - As cobras da região leste do Pará. *Publ. Avul. Mus. Par. Emílio Goeldi* **31**, 1-218.

Darin KA, Fox GM. 2002 *Xenopeltis unicolor*. Diet. *Herp Rev* **33 (3)**, 216.

Downs FL. 1967 Intrageneric relationships among colubrid snakes of the genus *Geophis* Wagler. Misc. *Pub. Mus. Zool. Univ. Michigan* **131**, 1-193.

Duellman WE. 1978 The biology of an Equatorial herpetofauna of Amazonian Ecuador. *Misc. Publ. Univ. Kansas* **65**, 1-352.

Eniang EA, Akani GC, Rugiero L, Vignoli L, Luiselli L. 2013 Ecological data of Nigerian *Crotaphopeltis hotamboeia* (Colubridae) populations. *Herpetol. J.* **23 (1)**, 5-9.

Filippi E, Capula M, Luiselli L, Agrimi U.1996 The prey spectrum of *Natrix natrix* (Linnaeus, 1758) and *Natrix tessellata* (Laurenti, 1768) in sympatric populations. *Herpetozoa* **8 (3/4)**,155-164.

Fitch HS. 1960 Autecology of the copperhead. *Univ. Kansas Publ. Mus. Nat. Hist.* **13**, 85-288.

Fong A, Bignotte I, García K. 2013 Unsuccessful predation on the toad *Peltophryne peltocephala* (Bufonidae) by the Cuban snake *Tropidophis melanurus* (Tropidophiidae). *Herpetol. Notes* **6**, 73-75.

França FGR, Mesquita DO, Nogueira CC, Araújo AFB. 2008 Phylogeny and ecology determine morphological structure in a snake assemblage in the central Brazilian Cerrado. *Copeia* **2008 (1)**, 23-38.

Gaiarsa MP, Alencar LRV, Martins M. 2013 Natural history of Pseudoboine snakes. *Pap. Avul. Zool.* **53 (19)**, 261-283.

Gans C. 1974 *Biomechanics: an approach to vertebrate biology*. Philadelphia, USA: JP Lippincott.

Gans C, Fetcho JR. 1982 The Sri Lankan genus *Aspidura* (Serpentes, Reptilia, Colubridae). *Ann. Carnegie Mus.* **51(14)**: 271-316.

Gardner SA, Mendelson JR. 2003 Diet of the leaf-nosed snakes, *Phyllorhynchus*

(Squamata, Colubridae): squamate-egg specialists. *Southwest. Nat.* **484**, 550-556.

Gartlan JS, Struhsaker TT. 1971 Notes on the habits of the Calabar ground python (*Calabaria reinhardtii* Schlegel) in Cameroon, West Africa. *British J. Herpetol.* **4**, 201-202.

Gibbons JW, Dorcas ME. 2004 *North American Watersnakes: A Natural History*. Norman: University of Oklahoma Press.

Giraudo AR, Arzamendia V, Bellini GP, Bessa CA, Costanzo MB. 2014. Ecología de una gran serpiente sudamericana, *Hydrodynastes gigas* (Serpentes: Dipsadidae). *Rev Mex Biodivers* **85 (4)**, 1206-1216.

Goodyear SE, Pianka ER. 2008 Sympatric ecology of five species of fossorial snakes (Elapidae) in Western Australia. *J Herpetol* **42**, 279-285.

Greene HW. 1980 Evolutionary biology of the dwarf boas (Serpentes: Tropidophiidae). *Yearb. Am. Phil. Soc.* **1979**, 206-207.

Greene HW. 1983 Dietary correlates of the origin and radiation of snakes. *Am Zool* **23**, 431-441.

Greene HW. 1984 Feeding behavior and diet of the Eastern coral snake, *Micrurus fulvius*. In *Vertebrate ecology and systematics-a tribute to Henry S. Firch* (eds RA Seigel, LE Hunt, JL Knight, L Malaret, NL Zuschlag), pp. 147-161. Lawrence, USA: Museum of Natural History of Kansas.

Greene HW. 1989 Ecological, evolutionary, and conservation implications of feeding biology in Old World cat snakes, genus *Boiga* (Colubridae). *Proc Cal Acad Sci* **46 (8)**, 193-207.

Greene HW, Seib RL. 1983 *Micrurus nigrocinctus* (coral, coral snake, coralillo). In *Costa Rican Natural History* (ed DH Janzen), pp. 406-408. Chicago, USA: The

University of Chicago Press.

Haluska F, Alberch P. 1989. The cranial development of *Elaphe obsoleta* (Ophidia, Colubridae). *J Morphol* **178**,37–55.

Harlow P, Shine R. 1992 Food habits and reproductive biology of the pacific island boas *Candoia*. *J Herpetol* **26 (1)**, 60-66.

Hartmann PA, Marques OAV. 2005 Diet and habitat use of two sympatric species of *Philodryas* (Colubridae), in south Brazil. *Amphibia-Reptilia* **26**, 25-31.

Henderson RW. 1984 The diets of hispaniolan colubrid snakes. I. Introduction and prey genera. *Oecologia* **62 (2)**, 234-239.

Henderson RW. 1993a Foraging and Diet in West Indian *Corallus enydris* (Serpentes: Boidae). *J Herpetol* **27 (1)**, 24-28.

Henderson RW. 1993b On the diets of some arboreal boids. *Herpetol Nat Hist* **1**, 91-96.

Henderson RW, Pauers MJ. 2012 On the diets of neotropical treeboas (Squamata: Boidae: *Corallus*). *South Am J Herpetol* **7 (2)**, 172-180.

Henderson RW, Noeske-Hallin TA, Ottenwalder JA, Schwartz A. 1987 On the diet of the boa *Epicrates striatus* on Hispaniola, with notes on *Epicrates fordi* and *Epicrates gracilis*. *Amphibia-Reptilia* **8**, 251-258.

Henderson RW, Schwartz A, Noeske-Hallin TA. 1987 Food habits of three colubrid tree snakes (genus *Uromacer*) on Hispaniola. *Herpetologica* **43 (2)**, 241-248.

Hofer AM, Auliya M. 2000 Observations on abundance and diet of the sunbeam snake *Xenopeltis unicolor* in west Kalimantan, Indonesia. *Sarawak Mus J* **55(76)**, 255-258.

Holm PA. 2008 Phylogenetic biology of the burrowing snake tribe Sonorini (Colubridae). PhD dissertation, The University of Arizona.

Holycross AT, Mackessy SP. 2002 Variation in the diet of *Sistrurus catenatus* (Massasauga), with emphasis on *Sistrurus catenatus edwardsii* (desert Massasauga). *J Herpetol* **36**, 454-464.

Houston D, Shine R. 1993 Sexual dimorphism and niche divergence: feeding habits of the Arafura filesnake. *J Anim Ecol* **62 (4)**, 737-748.

Hoyer RF, Stewart GR. 2000 Biology of the rubber boa (*Charina bottae*), with emphasis on *C. b. umbratica*. Part II: Diet, antagonists, and predators. *J Herpetol* **34 (3)**, 354-360.

Ineich I, Bonnet X, Shine R, Shine T, Brischoux F, Lebreton M, Chirio L. 2006 What, if anything, is a 'typical' viper? Biological attributes of basal viperid snakes genus *Causus* Wagler, 1830. *Biol J Linn Soc* **89 (4)**, 575-588.

Inger RF, Marx H. 1965 The systematics and evolution of the Oriental colubrid snakes of the genus *Calamaria*. *Fieldiana Zool* **49**, 1-304.

Jerez A. 2007. *Desarrollo del plan estructural de* Mabuya mabouya. PhD dissertation, Universidad Nacional de Tucumán, 175 p.

Johnson RG. 1955 The adaptive and phylogenetic significance of vertebral form in snakes. *Evolution* **9 (4)**, 367-388.

Keogh JS, Branch WR, Shine R. 2000 Feeding ecology, reproduction and sexual dimorphism in the southern African colubrid snake *Crotaphopeltis hotamboeia*. *Afr J Herpetol* **49**, 129-137.

Kluge AG. 1989 A concern for evidence and a phylogenetic hypothesis of relationships among *Epicrates* (Boidae: Serpentes). *Syst Zool* 38, 7-25.

Kupfer A, Gower DJ, Himstedt W. 2003 Field observations on the predation of the caecilian amphibian *Ichthyophis* sp. by the red-tailed pipe snake *Cylindrophis ruffus*. *Amphibia–Reptilia* **24**, 212-215.

Lee MSY, Caldwell MW. 1998 Anatomy and relationships of *Pachyrhachis problematicus*, a primitive snake with limbs. *Phil. Trans. Roy. Soc. London* B **353**, 1521-1552.

Leite PT, Nunes SF, Cechin SZ. 2007. Dieta e uso de habitat da jararaca-do-brejo, *Mastigodryas bifossatus* Raddi (Serpentes, Colubridade) em domínio subtropical do Brasil. *Rev Bras Zool* **24 (3)**, 729-734.

Leite PT, Kaefer IL, Cechin SZ. 2009 Diet of *Philodryas olfersii* (Serpentes, Colubridae) during hydroelectric dam flooding in southern Brazil. *Northwest J Zool* **5 (1)**, 53-60.

Lema T. 1989 Notas sobre a biologia de duas especies de *Elapomorphus* Wiegmann 1843 (Serpentes, Colubridae, Elapomorphinae). *Iheringia (Zool)* **69**, 61-69.

Lema T. 2001 Fossorial snake genus *Apostolepis* from South America (Serpentes: Colubridae: Elapomorphinae). *Cuad Herpetol* **15**, 29–43.

Leviton AE. 1964. Contributions to a review of Philippine snakes, III. The genera *Maticora* and *Calliophis*. *Philippine Journal of Science* **92**: 523-550.

Lillywhite HB, Henderson RW. 1993. Behavioral and functional ecology of arboreal snakes. In *Snakes: ecology and behavior*: 1-48. Seigel RA and Collins JT (Eds). New York: McGraw-Hill.

Lin CF, Tu MC. 2008 Food habits of the Taiwanese mountain pitviper, *Trimeresurus gracilis*. *Zool Stud* **47(6)**, 697-703.

Lindell LE. 1994 The evolution of vertebral number and body size in snakes. *Funct Ecol* **1994 (8)**, 708-719.

Lloyd CNV. 1974 Feeding behaviour in the green mamba *Dendroaspis angusticeps*. *J Herpetol Assoc Afr* **12**, 12-16.

López MS, Giraudo AR. 2004 Diet of the large water snake *Hydrodynastes gigas* (Colubridae) from northeast Argentina. *Amphibia-Reptilia* **25**, 178-184.

Lopez MS, Giraudo AR. 2008 Ecology of the Snake *Philodryas patagoniensis* (Serpentes, Colubridae) from Northeast Argentina. *J Herpetol* **42 (3)**, 474-480.

Luiselli L, Angelici FM. 1998 Sexual size dimorphism and natural history traits are correlated with intersexual dietary divergence in royal pythons (*Python regius*) from the rainforests of southeastern Nigeria. *Italian J Zool* **65**, 183-185.

Luiselli L, Akani GC. 2003 Diet of sympatric Gaboon vipers *Bitis gabonica* and nose-horned vipers *Bitis nasicornis* in southern Nigeria. *Afr J Herpetol* **52 (2)**, 101-106.

Luiselli L, Akani GC, Angelici FM. 2000. Arboreal habits and viper biology in the African rainforest: The ecology of *Atheris squamigera*. *Israel J Zool* **46 (4)**, 273-286.

Luiselli L, Angelici FM, Akani GC. 2000 Large elapids and arboreality: the ecology of Jameson's green mamba *Dendroaspis jamesoni* in an Afrotropical forested region. *Contr Zool* **693**, 147-155

Luiselli L, Akani GC, Capizzi D. 1998 Food resource partitioning of a community of snakes in a swamp rainforest of south-eastern Nigeria. *J Zool* **246**, 125-133.

Luiselli L, Angelici FM, Akani GC. 2002 Comparative feeding strategies and dietary plasticity of sympatric cobras *Naja melanoleuca* and *Naja nigricollis* in three diverging afrotropical habitats. *Can J Zool* **80 (1)**, 55-63.

Maddison, W. P. & Maddison, D. R. 2007. Mesquite: a modular system for evolutionary analysis. Version 2.0 http://mesquiteproject.org

Marques OAV. 2002 Natural history of the coral snake *Micrurus decoratus* (Elapidae) from the Atlantic Forest in southeast Brazil, with comments on possible mimicry. *Amphibia-Reptilia* **23 (2)**, 228-232.

Marques OAV, Puorto G. 1998 Feeding, reproduction and growth in the crowned snake *Tantilla melanocephala* (Colubridae) from southeastern Brazil. *Amphibia-Reptilia* **19**, 311-318.

Marques OAV, Sazima I. 1997 Diet and feeding behavior of the coral snake, *Micrurus corallinus*, from the Atlantic forest of Brazil. *Herpetol Nat Hist* **5 (1)**, 88-93.

Martins M, Oliveira ME. 1993 The snakes of the genus *Atractus* Wagler. (Reptilia: Squamata: Colubridae) from the Manaus region, central Amazonia, Brazil. *Zool Meded* **67 (1-26)**, 21-40.

Martins M, Oliveira ME. 1998 Natural history of snakes in forests of the Manaus region, Central Amazonia, Brazil. *Herpetol Nat Hist* **6**, 78-150.

Martins BH, Rosa GM. 2012 *Xenopeltis unicolor* Boie, 1827 predation upon *Sphenomorphus* sp. *Taprobanica* **4 (1)**, 48-51.

Martins M, Marques OAV, Sazima I. 2002 Ecological and phylogenetic correlates of feeding habits in Neotropical pitvipers of the genus *Bothrops*. In *Biology of the Vipers* (eds GW Schuett, M Hoggren, HW Greene), pp. 307-328. Eagle Mountain, USA: Eagle Mountain Publishing.

Marx H, Rabb GB. 1972 Phyletic analysis of fifty characters of advanced snakes. *Fieldiana (Zool)* **63**, 1-321.

Maschio GF, Prudente ALC, Rodriguez FS, Hoogmoed MS. 2010 Food habits of *Anilius scytale* (Serpentes: Aniliidae) in the brazilian Amazonia. *Zoologia* **27 (2)**, 184-190.

McDonald PJ, Luck GW, Wassens S, Pavey CR. 2011 Ecology of Stimson's python (*Antaresia stimsoni*) in the MacDonnell Ranges of central Australia. *Austr J Zool* **59**, 95-102.

Mora JM. 1987 Predation by *Loxocemus bicolor* on the eggs of *Ctenosaura similis* and *Iguana iguana*. *J Herpetol* **21 (4)**, 334-335.

Mora JM. 1991 *Loxocemus bicolor* (burrowing python). Feeding behavior. *Herp Rev* **22 (2)**, 61.

Mora JM, Robinson DC. 1984 Predation of sea turtle eggs *(Lepidochelys)* by the snake *Loxocemus bicolor* Cope. *Rev Biol Trop* **32**, 161-162.

Mushinsky HR, Hebrard JJ. 1977 Food partitioning by five species of water snakes in Louisiana. *Herpetologica* **33**,162-166.

Oliveira RB, Di-Bernardo M, Funk Pontes GM, Maciel AP, Krause L. 2001 Dieta e comportamento alimentar da cobranariguda, *Lystrophis dorbignyi* (Duméril, Bibron & Duméril, 1854), no litoral norte do Rio Grande do Sul, Brasil. *Cuad Herpetol* **14 (2)**, 117-122.

Orlov NL. 2000 Distribution, biology and comparative morphology of the snakes of *Xenopeltis* genus (Serpentes: Macrostomata: Xenopeltidae) in Vietnam. *Russ J Herpetol* **7 (2)**, 103-114.

Parpinelli L, Marques OAV. 2015. Reproductive biology and food habits of the blindsnake *Liotyphlops beui* (Scolecophidia: Anomalepididae). *South American Journal of Herpetology* **10 (3)**, 205-210.

Pendlebury GB. 1974 Stomach and intestine contents of *Corallus enydris*; a comparison of island and mainland specimens. *J Herpetol* **8**, 241-244.

Pinto RP, Fernandes R, Marques OAV. 2008 Morphology and diet of two sympatric colubrid snakes, *Chironius flavolineatus* and *Chironius quadricarinatus*

(Serpentes: Colubridae). *Amphibia-Reptilia* **29**, 149-160.

Pizzatto L, Marques OAV, Facure K. 2009 Food habits of Brazilian boid snakes: overview and new data, with special reference to *Corallus hortulanus*. *Amphibia-Reptilia* **30 (4)**, 533-544.

Plummer MV, Goy JM. 1984 Ontogenetic dietary shift of water snakes (*Nerodia rhombifera*) in a fish hatchery. *Copeia* **1984 (2)**, 550-552.

Prieto YA, Giraudo AR, Lopez MS. 2012 Diet and sexual dimorphism of *Liophis poecilogyrus* (Serpentes, Dipsadidae) from the wetland regions of Northeast Argentina. *J Herpetol* **46 (3)**, 402-406.

Prudente ALC, Menks AC, da Silva FM, Maschio GF. 2014 Diet and reproduction of the western indigo snake *Drymarchon corais* (serpentes: Colubridae) from the Brazilian Amazon. *Herpetol Notes* **7**, 99-108.

Puente-Rolón AR, Bird-Picó FJ. 2004 Foraging behavior, home range, movements and activity patterns of *Epicrates inornatus (*Boidae*)* at Mata de Plátano Reserve in Arecibo, Puerto Rico. *Carib J Sci* **40**, 343-352.

Punzo F. 1974 Comparative analysis of the feeding habits of two species of Arizona blind snakes, *Leptotyphlops h. humilis* and *Leptotyphlops d. dulcis*. *J Herpetol* **8**, 153-156.

Pyron RA, Burbrink FT, Colli GR, Montes de Oca AN, Vitt LJ, Kuczynski CA, Wiens JJ. 2011. The phylogeny of advanced snakes (Colubroidea), with discovery of a new subfamily and comparison of support methods for likelihood trees. *Molecular Phylogenetics and Evolution* **58**: 329-342.

Pyron RA, Kandambi HKD, Hendry CR, Pushpamal V, Burbrink FT, Somaweera R. 2013. Genus-level molecular phylogeny of snakes reveals the origins of species richness in Sri Lanka. *Molecular Phylogenetics and Evolution* **66**: 969-978.

Rage J-C, Escuillié F. 2000 Un nouveau serpent bipède du Cénomanien (Crétacé). Implications phylétiques. *CR. Acad. Sci.* **330**, 513-520.

Rajendran MV. 1985 *Studies in uropeltid snakes*. Madurai, India: Madurai Kamaraj University.

Ribeiro LC. 2006 *Phalotris mertensi* (False Coral Snake) and *Amphisbaena mertensi*. Predation. *Herp Rev* **37 (2)**, 234.

Rieppel O. 1994. Studies on skeleton formation in reptiles. III. Patterns of ossification in the skeleton of *Lacerta agilis exigua* Eichwald (Reptilia, Squamata). *J Herpetol* 28 (2): 145-153.

Rieppel O, Head JJ. 2004 New specimens of the fossil snake genus *Eupodophis* Rage & Escuillié, from Cenomanian (Late Cretaceous) of Lebanon. *Mem. Soc. Ital. Sci. Nat.* **32 (2)**, 1-26.

Rieppel O, H Zaher, E Tchernov & MJ Polcyn. 2003 The anatomy and relationships of *Haasiophis terrasanctus*, a fossil snake with well developed hind limbs from the mid-Cretaceous of the Middle East. *J. Paleont.* **77 (3)**, 536-558.

Rivas JA. 2000 The life history of the green anaconda (*Eunectes murinus*), with emphasis on its reproductive biology. PhD dissertation, The University of Tennessee, Knoxville.

Rodríguez-Robles JA, Greene HW. 1999 Food habits of the Long-nosed Snake (*Rhinocheilus lecontei*), a "specialist" predator? *J Zool* **248 (4)**, 489-499.

Rodríguez-Robles JA, Bell, CJ, Greene HW. 1999 Food habits of the glossy snake, *Arizona elegans*, with comparisons to the diet of sympatric long-nosed snakes, *Rhinocheilus lecontei*. *J Herpetol* **33**, 87-92.

Rodríguez-Robles JA, Mulcahy DG, Greene HW. 1999. Feeding ecology of the Desert Nightsnake, *Hypsiglena torquata* (Colubridae). *Copeia* **1999 (1)**, 93-100.

Roze AJ. 1982 New World coral snakes (Elapidae): a taxonomic and biological summary. *Mem Inst Butantan* **46**, 305-338.

Ruffato R, Di-Bernardo M, Maschio GF. 2003 Dieta de *Thamnodynastes strigatus* (Serpentes, Colubridae) no sul do Brasil. *Phyllomedusa* **2 (1)**, 27-34

Saint-Girons H. 1972 Les Serpents du Cambodge. *Mem Mus Nat D 'Hist Nat 5er. A,* **74**, 1-170.

Sant'Anna SS, Abe AS. 2007 Diet of the rattlesnake *Crotalus durissus* in southeastern Brazil (Serpentes, Viperidae), *Stud Neotrop Fauna Environ* **42**, 169-174.

Savitzky AH. 1978 The *origin* of the *New World proteroglyphous snakes* and *its bearing* on the *study* of *venom delivery systems* in *snakes.* PhD dissertation, University of Kansas, Lawrence.

Savitzky AH. 1983 Coadapted character complexes among snakes: fossoriality, piscivory, and durophagy. *Am. Zool.* **23**, 397-409.

Savitzky BAC. 1989 Aquatic foraging in two independently evolved species of snake: *Nerodia rhombifer* (Colubridae) and *Agkistrodon piscivorus* (Viperidae). PhD dissertation, University of Tennessee, Knoxville.

Sawaya RJ, Marques OAV, Martins M. 2008 Composition and natural history of a Cerrado snake assemblage at Itirapina, São Paulo state, southeastern Brazil. *Biota Neotrop* **8 (2)**, 127-149.

Scartozzoni RR, Salomão MG, Almeida-Santos SM. 2009 Natural history of the vine snake *Oxybelis fulgidus* (Serpentes, Colubridae) from Brazil. *South Am J Herpetol* **4 (1)**, 81-89.

Schwartz A, Henderson RW. 1991 *Amphibians and reptiles of the West Indies. Descriptions, distributions, and natural history*. Gainesville, USA: University of Florida Press.

Seib RL. 1985 Feeding ecology and organization of Neotropical snake faunas. PhD Dissertation. University of California, Berkley. 229pp.

Seib RL. 1985 Euryphagy in a tropical snake, *Coniophanes fissidens*. *Biotropica* **171**, 57-64.

Shine R. 1980a Comparative ecology of three australian snake species of the genus *Cacophis* (Serpentes: Elapidae). *Copeia*, **1980 (4)**, 831-838.

Shine R. 1980b Reproduction, feeding and growth in the Australian burrowing snake *Vermicella annulata*. *J Herpetol* **14 (1)**, 71-77.

Shine R. 1980c Ecology of the Australian death adder, *Acanthophis antarcticus* (Elapidae): evidence for convergence with the Viperidae. *Herpetologica* **36**, 281-289.

Shine R. 1984 Ecology of small, fossorial Australian snakes of the genera *Neelaps* and *Simoselaps* (Serpentes: Elapidae). In *Vertebrate ecology and systematics-a tribute to Henry S. Firch* (eds RA Seigel, LE Hunt, JL Knight, L Malaret, NL Zuschlag), pp. 173-183. Lawrence, USA: Museum of Natural History of Kansas.

Shine R. 1986 Ecology of a low energy specialist: food habits and reproductive biology of the Arafura filesnake (Acrochordidae). *Copeia* **1986**, 424-437.

Shine R. 1991 Strangers in a strange land: ecology of the Australian colubrid snakes. *Copeia* **1991**, 120-131.

Shine R, Covacevich J. 1983 Ecology of highly venomous snakes: the Australian genus *Oxyuranus* (Elapidae). *J Herpetol* **17 (1)**, 60-69.

Shine R, Slip DJ. 1990 Biological aspects of the adaptive radiation of Australasian pythons (Serpentes: Boidae). *Herpetologica* **46**, 283-290.

Shine R, Webb JK. 1990 Natural history of Australian typhlopid snakes. *J Herpetol* **24**, 357-363.

Shine R, Keogh JS. 1996 Food habits and reproductive biology of the endemic Melanesian elapids: are tropical snakes really different? *J Herpetol* **30**, 238-247.

Shine R, Wall M. 2008 Interactions between locomotion, feeding, and bodily elongation during the evolution of snakes. *Biol J Linn Soc* **95**, 293-304.

Shine R, Ambariyanto L, Harlow PS, Mumpuni P. 1999 Reticulated pythons in Sumatra: biology, harvesting and sustainability. *Biol Conserv* **87**, 349-357.

Shine R, Spencer CL, Keogh JS. 2014 Morphology, Reproduction and Diet in Australian and Papuan Death Adders (*Acanthophis*, Elapidae). PLoS ONE 9(4): e94216. doi:10.1371/journal.pone.0094216

Shine R, Branch WR, Harlow PS, Webb JK. 1996a Life on the lowest branch: sexual dimorphism, diet and reproductive biology of an African twig snake, *Thelotornis capensis* (Serpentes, Colubridae). *Copeia* **1996**, 290-299.

Shine R, Branch WR, Harlow PS, Webb JK. 1996b Sexual dimorphism, reproductive biology and food habits of two species of African filesnakes (*Mehelya*, Colubridae). *J Zool* **240**, 327-340.

Shine R, Harlow PS, Keogh JS, Boeadi K. 1998 The influence of sex and body size on food habits of a giant tropical snake, *Python reticulatus*. *Funct Ecol* **1998**, 248-258.

Shine R, Branch WR, Harlow PS, Webb JK. 1998 Reproductive biology and food habits of horned adders*, Bitis caudalis (*Viperidae), from southern Africa. *Copeia* **1998**, 391-401.

Shine R, Branch WR, Harlow PS, Webb JK, Shine T. 2006a Biology of burrowing Asps (Atractaspididae) from Southern Africa. *Copeia* **2006 (1)**, 103-115.

Shine R, Branch WR, Harlow PS, Webb JK, Shine T. 2006b Sexual dimorphism, reproductive biology and dietary habits of psammophiine snakes (Colubridae) from southern Africa. *Copeia* **2006**, 650-664.

Sironi M, Chiaraviglio M, Cervantes R, Bertona M, Rio M. 2000 Dietary habits of *Boa constrictor occidentalis*, in the Cordoba Province, Argentina. *Amphibia-Reptilia* **21**, 226-232.

Slip DJ, Shine R. 1988 Feeding habits of the diamond python, *Morelia* s. *spilota:* Ambush Predation by a boid snake. *J Herpetol* **22 (3)**, 323-330.

Smith GR, Lemos-Espinal A, Dibble CJ, Iverson JP. 2008 Natural History of *Procinura aemula* (Serpentes: Colubridae) From Chínipas, Chihuahua, Mexico. *Southwest Nat* **53 (2)**, 261-264.

Souza SM, Junqueira AB, Conte Jakovac AC, Apostolo P, Adailton CJ. 2011 Feeding behavior and ophiophagous habits of two poorly known amazonian coral snakes, *Micrurus albicinctus* Amaral 1926 and *Micrurus paraensis* Cunha and Nascimento 1973 Squamata, Elapidae. *Herpetol Notes* **4**, 369-372.

Strüssmann C. 1997 Hábitos alimentares da sucurí-amarela, *Eunectes notaeus* Cope, 1862, no Pantanal matogrossense. *Biociencias* **5**, 35-52.

Vinson J. 1949 L'i'le Ronde et aux Serpents. *Proc. R. Soc. Arts Sci. Mauritius* **1**, 32-52.

Tarazona OA, Fabrezi M, Ramirez-Pinilla MA. 2008. Cranial morphology of *Bachia bicolor* (Squamata, Gymnophtalmidae) and its postnatal development. *Zool J Linn Soc* **152**, 775-792.

Taylor EN. 2001 Diet of the Baja California rattlesnake, *Crotalus enyo* (Viperidae). *Copeia* **2001(2)**, 553–555.

Tchernov E, Rieppel O, Zaher H, Polcyn MJ & Jacobs IJ. 2000. A new fossil snake

with limbs. *Science* **287**, 2010–2012.

Vanzolini PE. 1970 Climbing habits of Leptotyphlopidae (Serpentes) and Wall's theory of the evolution of the ophidian eye. *Pap Av Zool*, **23**, 13-16.

Vincent SE, Herrel A, Irschick DJ. 2004a Ontogeny of intersexual head shape and prey selection in the pitviper Agkistrodon piscivorus. *Biol J Linn Soc* **81**, 151-159.

Vincent SE, Herrel A, Irschick DJ. 2004b Sexual dimorphism in head shape and diet in the cottonmouth snake (*Agkistrodon piscivorus*). *J Zool* **264**, 53-59.

Vinson J, Vinson J-M. 1969 The saurian fauna of the Mascarene Islands. I. A revision of the fauna. *Bull. Mauril. Ins.* **6**, 203-320.

Vitt LJ, Hulse AC. 1973 Observations on feeding habits and tail display of the sonoran coral snake, *Micruroides euryxanthus*. *Herpetologica* **29 (4)**, 302-304.

Vitt LJ, Vangilder LD. 1983. Ecology of a snake community in Northeastern Brazil. *Amphibia-Reptilia* **4**: 273-296.

Voris HK. 1966 Fish eggs as the apparent sole food item for a genus of sea snake, *Emydocephalus* (Krefft). *Ecology* **47**, 152-154

Voris HK, Glodek GS. 1980 Habitat, diet, and reproduction of the file snake, *Acrochordus granulatus*, in the straits of Malacca. *J Herpetol* **14 (1)**, 108-111.

Voris HK, Voris HH. 1983. Feeding strategies in marine snakes: an analysis on evolutionary, morphological, behavioral and ecological relationships. *Am Zool* **23**, 411-425.

Wallace RL, Diller LV. 1990 Feeding ecology of the rattlesnake, *Crotalus viridis oreganus*, in northern Idaho. *J Herpetol* **24 (3)**, 246-253.

Waller T, Buongermini P, Micucci PA. 2001 *Eunectes notaeus* (Yellow Anaconda): diet. *Herp Rev* **32**, 47.

Waller T, Micucci PA, Alvarenga E. 2007 Conservation biology of the Yellow Anaconda (*Eunectes notaeus*) in Northeastern Argentina. In *Biology of the Boas and Pythons* (eds R Henderson, R Powell), pp. 340-362. Eagle Mountain, USA: Eagle Mountain Publishing.

Webb JK, Shine R, Branch WR, Harlow PS. 2000 Life history strategies in basal snakes: reproduction and dietary habits of the African threadsnake, *Leptotyphlops scutifrons* (Serpentes, Leptotyphlopidae). *J Zool* **250**, 321-327.

Webb JK, Shine R, Branch WR. 2001 Dietary habits and reproductive biology of typhlopid snakes from southern Africa. *J Herpetol* **35**, 558-567.

Weiperth A, Gaebele T, Potyó I, Puky M. 2014 A global overview on the diet of the dice snake (*Natrix tessellata*) from a geographical perspective: foraging in atypical habitats and feeding spectrum widening helps colonisation and survival under suboptimal conditions for a piscivorous snake. *Zool Stud* **53**, 1-9.

Wiley JW. 2003 Habitat association, size, stomach contents, and reproductive condition of Puerto Rican Boas (*Epicrates inornatus*). *Carib J Sci* **39**, 189-194.

Wilson D. 2007 Foraging ecology and diet of an ambush predator: the Green Python (*Morelia viridis*). In *Biology of the Boas and Pythons* (eds R Henderson, R Powell), pp. 141-150. Eagle Mountain, USA: Eagle Mountain Publishing.

Witberg M, Van Zyl G. 2006 *Homoroselaps lacteus*. Diet/predation. *Afr Herp News* **40**, 21.
